# Vitamin deficiencies and Alzheimer’s disease: evidence and implications for supplementation

**DOI:** 10.3389/fnut.2026.1676497

**Published:** 2026-02-13

**Authors:** Mariya Timotey Miteva, Davide Laurenti, Roberto Mattioli, Daniel Di Risola, Alessia Mariano, Luciana Mosca

**Affiliations:** 1 Institute of Translational Pharmacology, CNR, Rome, Italy; 2 Department of Public Health and Infectious Diseases, Sapienza University of Rome, Laboratory Affiliated to Istituto Pasteur Italia-Fondazione Cenci Bolognetti, Rome, Italy; 3 Department of Biochemical Sciences, Faculty of Pharmacy and Medicine, Sapienza University, Rome, Italy

**Keywords:** Alzheimer’s disease, hypovitaminosis, neurodegeneration, nutrition, oxidative stress, vitamin supplementation, vitamin cocktail, vitamins

## Abstract

Alzheimer’s disease (AD) is a progressive neurodegenerative disorder characterized by beta-amyloid (Aβ) deposition, hyperphosphorylation of tau protein (pTau), mitochondrial impairment and neuroinflammation. Several risk factors, such as aging, genetics, cardiovascular diseases (CVD) and lifestyle, concur to the onset of the disease. Among modifiable risk factors, micronutrient intake has gained attention for its potential role in preventing or slowing down disease progression. In this narrative review, we summarize current evidence linking vitamin deficiencies to the onset and progression of AD. We analyze evidence for fat-soluble (A, D, E, K) and water-soluble vitamins (C and B-complex, both canonical B1–B12 and non-canonical forms such as B13, B15, and B17). We then analyze individual and combinational vitamin supplementation in AD patients as the primary focus, with additional data derived from animal and cellular studies when human data are limited. As final result, B6, B9, and B12 vitamins have demonstrated promising effects in clinical trials. Interestingly, some beneficial effects have also been observed in the prodromal stage of AD when these vitamins were combined with antioxidant compounds such as vitamin C and vitamin E. Given the multifactorial nature of AD, targeting isolated vitamin deficiencies may not be sufficient. Future research should focus on long-term clinical trials (at least 2 years), particularly exploring combinations of vitamins and antioxidants, to achieve meaningful therapeutic effects. This review is intended as a point of support for future clinical trials in the treatment of AD from a nutritional point of view.

## 1 Introduction

More than a century after the description of the first AD patient and the realization that only a small percentage (~ 1%) of AD patients can be traced back to the genetic hypothesis (1), the scientific community still wonders what factors predispose to the onset and progression of AD. To date, numerous hypotheses on the etiopathogenesis of this disease have been proposed. These range from the classic hypotheses linking it to Aβ and pTau, to those implicating cholinergic and calcium signaling. Other hypotheses point to neuroinflammation, oxidative stress, and mitochondrial dysfunction as the culprits of the disease (2). Although there is no unified hypothesis, it is certain that the factors impacting AD onset are manifold: they can range from diet to metabolic diseases (such as diabetes and CVD) (3), infections (4), brain injuries (5) to toxicity from metals and PM 2.5 (5, 6) and many others. As long as the causes for AD onset are not fully elucidated, the best strategy to reduce the risk is to act on modifiable risk factors. Among them, diet can provide the body with useful molecules that can counteract oxidative stress, inflammation and tissue ageing (7–10), preserving the nervous system. Beyond the main nutrients, there are some important micronutrients that possess neuroprotective effects, such as vitamins, minerals, polyunsaturated fatty acids (PUFAs) and polyphenols (11).

In AD patients, vitamin deficiencies are typical phenomena. However, it is unclear whether vitamin and nutritional deficiencies actually contribute to AD progression or whether the disease itself causes nutritional deficiencies as it can lead to changes in diet and lifestyle. For instance, it should be considered that the activity of the mesial temporal cortex, involved in the control of memory and food intake in the central nervous system (CNS), is functionally impaired in dementia. This causes an alteration in serotoninergic, dopaminergic and adrenergic neurotransmission, which in turn are involved in regulating eating behavior and leading to loss of appetite and refusal to eat, decreasing food intake (12, 13). On the other hand, vitamins serve numerous vital functions including cognitive function (14). Therefore, a deficiency of these micronutrients may aggravate the loss of cognitive activity or even aggravate dementia, creating a vicious circle in these patients.

**Figure 1:**
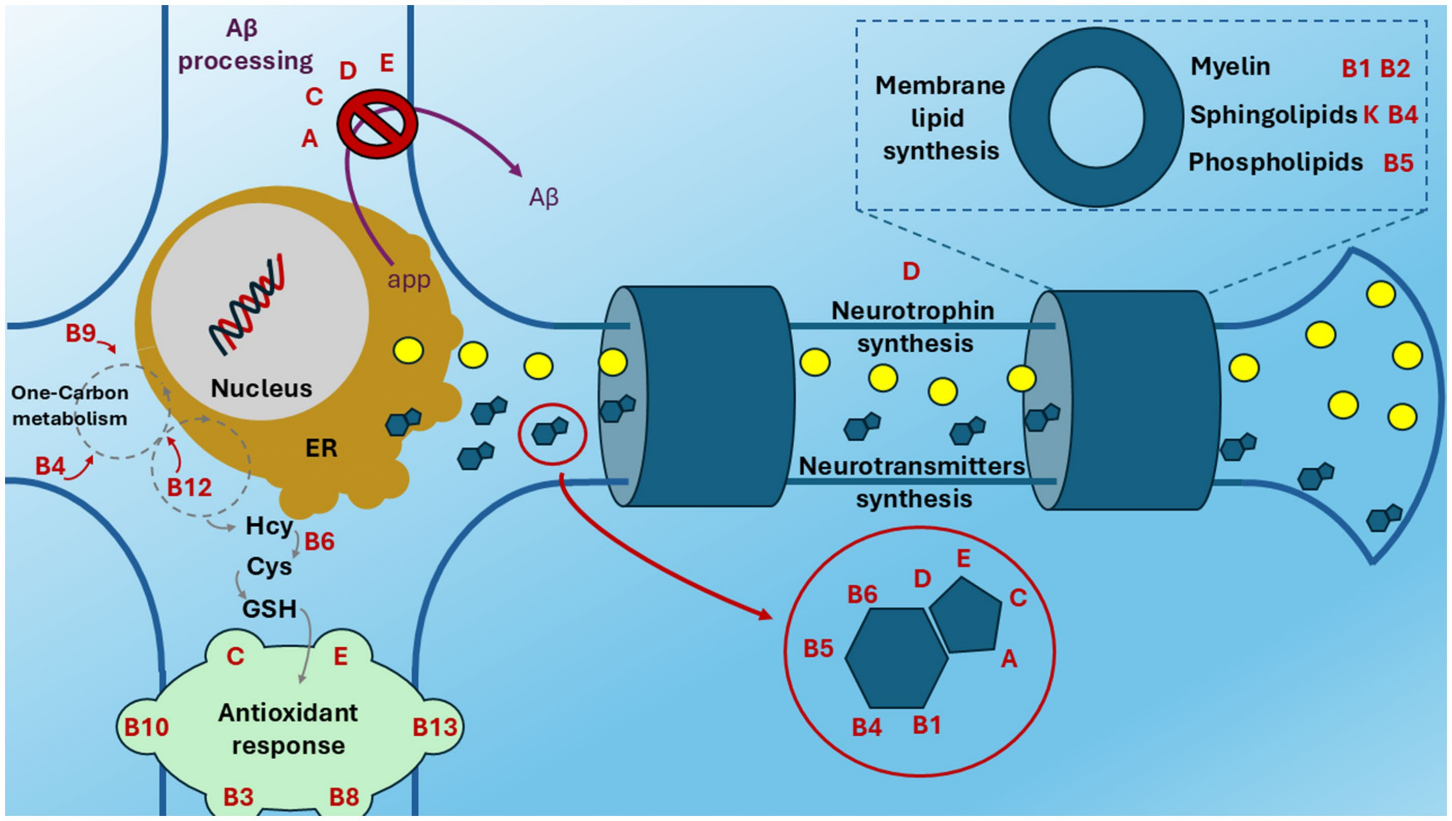
Vitamin involvement in neuronal physiology. The main vitamin-linked processes in neuronal physiology (neurotrophin, neurotransmitters and membrane lipid synthesis, antioxidant response) are indicated in bold black. Aβ processing is indicated in bold purple. Vitamins (bold red) are near to the process they are involved in. This figure is based on the references summarised in Supplementary Tables S1, S2. ER, endoplasmic reticulum; Aβ: beta-amyloid; Hcy, homocysteine; Cys, cysteine; GSH, glutathione.

In recent years, increasing attention was put on the correct nutrition and vitamin intake in AD patients. The evaluation of serum vitamin levels is being considered for a proper supplemental therapy in combination with traditional drugs and, in some cases, as an additional diagnostic marker in the early stages (15, 16). A summary of the known activities of vitamins in the nervous system is provided in Supplementary Tables S1, S2. Thus, the aim of this narrative review is to give an overview of how vitamin deficiencies are related to AD and which studies are being conducted in order to restore a proper intake of these micronutrients to counteract AD. This review is divided into three sections: the first deals with vitamin deficiencies in nervous system, the second with vitamin supplementation on patients and in preclinical models, the third with a particular focus on the use of vitamin cocktails. Figure 1 shows the main neuronal physiological pathways in which the vitamins are involved.

## 2 Search strategy

For the purposes of writing this Review, articles published on PubMed were used with regard to preclinical and clinical studies. The keywords ‘vitamin ()’ or other name of the vitamin (common, chemical) alone or + ‘AD’ or ‘Alzheimer’ were used, and the articles most relevant to the topic and most rigorous and comprehensive from a scientific point of view were selected, without time limitations.

Clinical studies were searched for on ClinicalTrial.gov using the keywords “vitamin ()” or other name of the vitamin (common, chemical) + “AD” or “Alzheimer’s.” All relevant clinical studies were reported, without time limitations. The tables concerning clinical trials show the sample size (n) of each experimental arm that reached the end of the trial and was analysed in the scientific paper.

## 3 Vitamin deficiencies

### 3.1 Fat-soluble vitamins

A comprehensive table summarizes the effects of lipophilic vitamins in the nervous system (Supplementary Table S1).

#### 3.1.1 Vitamin A

The term vitamin A refers to various fat-soluble substances, including retinol, retinoic acids (RA) and retinyl esters. These substances are essential for the body as they are involved in processes such as vision, cell differentiation, and immunity (17). In the brain, vitamin A is necessary for the control of genes involved in the growth, survival and differentiation of neuronal cells. Moreover, animal studies have highlighted that vitamin A has significant effects on brain physiology and behavior (18).

Considering the activities exerted by vitamin A, researchers investigated its involvement in AD pathogenesis (19). Evidence on rats has shown that a vitamin A deficiency (VAD) lasting 6 months decreases Retinoic Acid Receptors (RARs) activation and reduces the expression of choline acetyltransferase, the enzyme responsible for acetylcholine (ACh) synthesis (20, 21). Although no neuronal loss was reported, this phenomenon precedes the accumulation of Aβ plaques that occur after 1 year of VAD (20). This reduction in RARs has been observed already in the early stages of the disease in AD patients (22). Moreover, Almaguer et al. demonstrated that All-Trans-Retinoic-Acid (ATRA), acts as agonist of RARs, increasing the expression of α-secretase that prevents the Aβ accumulation (23). ATRA is a lipophilic molecule and can diffuse easily through the cellular membrane reaching the brain cells (24). For these characteristics, ATRA was considered as a novel therapeutic strategy for AD treatment, as an additional alternative to the vitamin A supplement. In this respect, an interesting study conducted on immortalized human microglia cells (CVCL_5G94) highlighted how ATRAs can increase the heat shock protein 90 (Hsp90) expression on the surface of these cells, causing an increase in the amount of endocytosed and consequently eliminated tau protein (25). These cells are macrophage-like cells and are involved in the brain’s innate immune system, whose defense mechanisms are altered in neurodegenerative diseases such as AD (26). These results were in line with a C57BL/6 J APP/PS1 transgenic mouse model, in which vitamin A deprivation leads to an increase in the cortex of pTau in addition to an increase in Aβ40 and Aβ42 in the cortex and in the expression of β-site amyloid precursor protein-cleaving enzyme 1 (BACE1) gene transcript (27). This protective role has been confirmed *in vitro* and *in vivo* on *Caenorhabditis elegans* (*C. elegans*). Joshi and colleagues demonstrated that RA is capable of inhibiting Aβ aggregation in a dose-dependent manner in the early stages of this process (28). Finally, many studies on rodent models report that VAD exacerbates Aβ deposition, tau phosphorylation, and pathological degeneration in AD animal models (29), but the exact mechanism still remains unknown.

In summary, Vitamin A appears to have significant effects on cellular management of Aβ and pTAU through its receptor, and its hypovitaminosis significantly impacts these parameters, promoting their aggregation.

#### 3.1.2 Vitamin D

Vitamin D, including D2 (ergocalciferol) and D3 (cholecalciferol), is a fat-soluble molecule responsible for the intestinal absorption of calcium, magnesium and phosphate, and it is involved in maintaining calcium homeostasis to ensure proper bone mineralization (30). Aside from its central role in calcium homeostasis and bone health, vitamin D has been shown to play a key role in the nervous system. It regulates differentiation, proliferation of neurons and microglia, and it is involved in the dopaminergic signaling pathway, affecting brain development and synaptic plasticity (31). Vitamin D also regulates neurotrophin release, including Nerve Growth- (NGF), Glial cell line-derived- (GDNF) and Brain-Derived Neurotrophic-Factors (BDNF), and the synthesis of some neurotransmitters, including ACh, dopamine (DA), 5-HydroxyTryptamine/serotonin (5-HT) and γ-aminobutyric acid (GABA) (32). The decrease of neurotrophins, in serum and in CNS (33) has been associated with the formation of Aβ and pTau plaques (34–36). Unfortunately, vitamin D level wasn’t assessed in these studies.

Recently, several researchers have questioned the role of vitamin D deficiency in AD pathogenesis. While some studies did not show any relationship between blood levels of vitamin D and AD symptoms (37, 38), others have reported a clear association. One of the first studies to suggest a link between vitamin D and AD dates back to 1992, when Sutherland et al. demonstrated a reduced expression of vitamin D receptors (VDRs) in the hippocampus of AD patients (39). Interestingly, it has been described that VDR expression can be modulated by vitamin D itself (40), thus demonstrating an involvement of both ligand and receptor downmodulation in AD. Since then, large-scale studies have been carried out. Among these, a longitudinal study of over half a million participants revealed strong associations between vitamin D serum level deficiency or insufficiency with AD (41). Moreover, a systematic review and meta-analysis conducted in 2023, analyzed five prospective studies and one cross-sectional study, involving 10,884 participants overall. The meta-analysis showed that patients with low serum vitamin D levels (<25 ng/mL) had a higher risk of developing AD than patients with normal vitamin D levels and that a severe vitamin D deficiency (< 10 ng/mL) was associated with an even higher risk (42). Further support for an association between vitamin D and cognitive impairment comes from recent population-based studies: for instance, an analysis of NHANES data showed that vitamin D deficiency in older adults is significantly associated with cognitive impairment (43).

However, although most studies indicate a relationship between reduced vitamin D levels and AD, some authors, including Chai et al., make clear that these kinds of analyses can be influenced by the reverse causality bias (44–46). Therefore, it is difficult to unequivocally determine whether low vitamin D levels are responsible for dementia and cognitive impairment typical of AD or if dementia can lead to reduced intake of vitamin D. Indeed, due to their loss of autonomy, patients suffering from AD or dementia spend less time outdoors or in sunlight, causing vitamin D deficiency over time.

In summary, Vitamin D has direct effects on the nervous system, mainly due to the synthesis of neurotrophins. Population studies have shown an increased incidence of developing AD when levels are low, although caution must be exercised with regard to reverse causality bias.

#### 3.1.3 Vitamin E

Vitamin E is a term comprising eight isoforms (vitamers) of micronutrients, specifically α-, β-, γ-, and δ-tocopherol, and α-, β-, γ-, and δ-tocotrienol. Among these, 𝛼-tocopherol is the dominant isoform (47). Vitamin E members, particularly tocopherols, are of great interest in AD because of their antioxidant properties. In the scavenging process free radicals form the stable radical of vitamin E, which is then subsequently reduced by vitamin C (48). Therefore, reduced vitamin E is active in inhibiting brain lipid peroxidation, further exerting its antioxidant activity (19, 49). However, the behavior of vitamin E within the context of the organism’s redox state is variable. Since vitamin E acts as an antioxidant through radical scavenging, it must be rapidly reduced by other antioxidants (e.g., vitamin C); otherwise, it can become pro-oxidant (50). In AD, oxidative stress plays an important role, indeed AD patients’ brains are more susceptible to oxidative stress because they have [Cu^2+^] and [Zn^2+^] levels three times higher than healthy brains. These metals can interact with the N-terminal portions of Aβ and generate a significant amount of reactive oxygen species (ROS). Additionally, there is a migration of Cu^2+^ from inside brain cells to Aβ plaques, thereby impairing the activity of metalloenzymes in brain cells (51).

Vitamin E deficiency affects a wide range of physiological pathways. Thus, in mouse and rat models it has been demonstrated that Vitamin E is responsible for the regulation of numerous genes essential for the elimination of Aβ, nerve cell growth, hormonal metabolism, apoptosis, and dopaminergic neurotransmission (52). A mouse model of AD showed that vitamin E depletion is responsible for the increase of Aβ plaques decreasing its clearance from brain and blood (53) thus enhancing AD pathology (54). These results are in line with some human and animal studies reported by Sultana et al. that show vitamin E deficiency can lead to cognitive impairment and a decrease in motor skills (18).

A study found a decrease in vitamers E (α-, γ-tocopherol and δ-tocotrienol) in plasma of AD patients compared with healthy ones. Moreover, a decrease, even if non statistically significant, in telomere length has been observed (55). Telomeres undergo shortening with each cell division and are sensitive to oxidative stress. Their involvement in AD pathogenesis has been discussed for decades and reported as associated with the aging process and aging-associated diseases (56). Casati et al. studied the connection between telomere length and vitamin E level by correlation analyses, showing an association between telomere length and γ-tocopherol, total tocopherols, and total vitamin E in AD patients (55).

Although all these studies agree that vitamin E levels correlate with AD, another important study highlights the importance of vitamin E aggregation state. Indeed, vitamin E is able to directly bind the aggregate state of Aβ in two ways *in vitro*. Aggregated forms of α-tocopherol can promote the aggregation of Aβ by acting as nucleation centers, conversely monomeric forms of α-tocopherol inhibit its aggregation (28).

In summary, Vitamin E exerts a direct effect as a ROS scavenger and Aβ interactor and an indirect effect in regulating Aβ elimination. Vitamin E deficiency has been linked to AD and cellular senescence.

#### 3.1.4 Vitamin K

Vitamin K refers to a group of fat-soluble compounds sharing a 2-methyl-1,4-naphthoquinone structure (menadione). They play key roles in blood clotting, bone metabolism, and antioxidant/anti-inflammatory functions, acting as cofactors in the γ-carboxylation of the glutamic acid residues of Vitamin K-dependent proteins (VKDPs) by activating them. Among these, Growth arrest-specific protein 6 (Gas6) and protein S1 (ProS1) are some of the Vitamin K protein targets found to be closely associated with brain health and cognitive functions (57–59). Indeed, they prevent cell apoptosis through the activation of the PI3K/Akt/Bad-signaling pathway (60). In addition to its better known activity, it has been shown that vitamin K has a fundamental role in sphingolipid synthesis, a class of lipids present in high concentrations in both neuronal and glial cell membranes (61, 62), and in inhibiting ferroptosis, a non-apoptotic form of cell death characterized by iron-dependent lipid peroxidation (63).

Although vitamin K deficiency is extremely rare in adults, its decrease in AD has to be considered with particular emphasis for its effects on the brain. In 2001, Allison and colleagues hypothesized that the link between low vitamin K levels and AD development may be related to the presence of the apolipoprotein E4 (APOE4) variant genotype in AD patients (64). It is now clear that APOE polymorphism represents a genetic risk factor for AD; but interestingly, it also exerts a key role in vitamin K absorption and clearance. APOE is a small protein which interacts with lipoprotein receptor, favoring the clearance of the chylomicrons, which are involved in vitamin K transport (65). This variant in AD patients is particularly effective in promoting vitamin K clearance resulting in a decrease in serum concentration (64, 66). An analysis performed on 320 subjects revealed an association between vitamin K status, measured as serum concentrations of phylloquinone, and cognitive impoverishment in the elderly. It was shown that patients with higher serum levels had better episodic verbal memory and memory track consolidation, compared to patients with lower levels (67). This result is in line with the study by Tamadon-Nejad et al. that demonstrated that in warfarin-induced vitamin K deficiency, rats have motor dysfunctions, slower learning acquisition and detrimental effect on cognition respect to the control group. Moreover, they found an altered expression and distribution of ganglioside, which is one of the major components of neuronal cell membranes, in key rat brain regions, suggesting a possible involvement of vitamin K in cognitive and behavioral perturbations (68).

In summary, Vitamin K is involved in the synthesis of sphingolipids and appears to be essential for the nervous system. Low levels of vitamin K may be linked to AD-related APOE variants.

### 3.2 Water-soluble vitamins

A comprehensive table summarizes the effects of hydrophilic vitamins in the nervous system (Supplementary Table S2).

#### 3.2.1 B vitamins

B vitamins are a group of water-soluble vitamins that play a key role in cell functioning, acting as coenzymes in many metabolic reactions. Their effect in the brain is particularly relevant for energy production, DNA synthesis and repair, methylation processes, as well as synthesis of numerous neurochemicals and signaling molecules (69). This group includes vitamins B1 (thiamine), B2 (riboflavin), B3 (niacin), B4 (choline), B5 (pantothenic acid), B6 (pyridoxine), B7 (biotin), B8 (inositols), B9 (folic acid), B10 (PABA), B12 (cobalamin), B13 (orotic acid), B15 (pangamic acid), and B17 (amygdalin). Thiamine, riboflavin, niacin, and pantothenic acid are essential co-enzymes in mitochondrial aerobic respiration and cellular energy production via their direct role in the Krebs cycle, the electron transport chain and the resultant formation of adenosine triphosphate (ATP) (70). Vitamin B members are also called “neurotropic” because of their essential role in the CNS (71).

In summary, many of these vitamins are involved in energy metabolism (such as B1, B2, B5), neurotransmitter synthesis (such as B6, B9), homocysteine management (B6, B9, B12), signalling (such as B8) and as precursors of useful molecules (B10 and B13). Of these, it is known that hypovitaminosis of B1 leads to oxidative stress, B3 to mitochondrial damage, and B12 to neurodegeneration. From vitamin B1 to vitamin B6, important studies on the correlation between their hypovitaminosis and AD are found in humans.

#### 3.2.2 Vitamin B1 (thiamine)

Vitamin B1 plays a key role in carbohydrate metabolism, ATP and NADPH synthesis and the generation of pentoses for nucleic acid biosynthesis. It is particularly important for the proper functioning of the heart, skeletal muscles and nervous system (72). In the brain, thiamine is present mainly as thiamine diphosphate (TDP), which represents an important cofactor for the pyruvate dehydrogenase complex (PDHC), transketolase (TK) and α-ketoglutarate dehydrogenase (α-KGDH). These enzymes are involved in energy metabolism and cellular glucose management. It is well established that the reduction of TDP concentration is linked to decrease of α-KGDH activity, which is in turn linked to metabolic defects responsible for neurological impairments, and to a decrease of TK activity (73, 74).

This vitamin is also involved in synapse formation and myelin genesis (75). Of note, low Vitamin B1 levels have also been associated with memory disorders and Aβ plaque formation (76). Indeed, in AD rodent models, the thiamine deficiency not only increases the glucose brain utilization, inflammation and neuronal loss, but also exacerbates plaque formation and tangles (77). Moreover, thiamine plays a critical role in ACh synthesis (73, 78)impacting the cholinergic system, which is profoundly impaired in AD (79).

Observational studies have examined whether alterations in thiamine status are associated with cognitive decline and AD. In a cross-sectional study of 206 patients with AD, Qian et al. evaluated blood B-vitamin status, nutritional markers and cognition, and identified TDP as the B-vitamin form most closely associated with both nutritional indices and cognitive performance. These data support the notion that reduced TDP may contribute to cognitive decline in AD, consistent with its central role in brain energy metabolism (80). In a 1:1 case–control study including 90 patients with AD and 90 cognitively normal controls, Wang et al. reported significantly lower whole-blood TDP levels in AD and an inverse association between TDP concentrations and the likelihood of having AD. They also observed lower TDP levels in female compared with male AD patients, suggesting that thiamine status may influence both overall AD risk and sex-related vulnerability (81).

#### 3.2.3 Vitamin B2 (riboflavin)

Vitamin B2 is known to be a precursor in the synthesis of both flavin mononucleotide (FMN), and flavin adenine dinucleotide (FAD), fundamental for the mitochondrial function, neuronal antioxidant response (82) and myelin synthesis (83). Riboflavin deficiency has been shown to influence iron absorption, tryptophan and other vitamin metabolism and mitochondrial function. It was reported that up to 60% of the population may be at risk of vitamin B2 deficiency (84). A study involving 368 AD patients and 574 controls was evaluated for serum vitamin levels and the authors concluded that lower serum vitamin B2, together with B9, B12, D and E, may be a risk factor for AD, unfortunately without proposing a possible molecular mechanism that could explain the correlation (85).

#### 3.2.4 Vitamin B3 (niacin)

Vitamin B3 is a precursor to produce nicotinamide adenine dinucleotide (NAD^+^) and nicotinamide adenine dinucleotide phosphate (NADP^+^) which plays a fundamental role in the neuronal energetic metabolism and participates in the antioxidant process and DNA repair (70). Niacin is involved in proper function of the immune system, and reduction of neuroinflammation, another important AD feature (86). Its deficiency has been linked to memory impairment, symptoms of dementia and mood changes (87). Moreover, NAD^+^ deficiency is associated with mitochondrial dysfunction which could be linked to AD (88, 89).

#### 3.2.5 Vitamin B4 (choline)

Vitamin B4, also known as choline, is an essential nutrient which plays a well-recognized role in brain function. It is necessary for the synthesis of ACh and choline-containing phospholipids, most of which are involved in cell membrane signaling. Additionally, choline plays a key role in One-carbon metabolism (OCM) (90). Studies in postmortem AD brains reveal changes in phospholipid metabolism. In fact, in the study by Nitsch and collaborators, the increased catabolism of membrane phospholipids in the AD brain was reported with a decrease of initial phospholipid precursor levels. All these data support the hypothesis that phospholipid turnover is elevated in neurodegeneration (91). Besides the CDP-choline pathway that depends on dietary choline, phosphatidylcholine (PC) can also be synthesized endogenously through phosphatidylethanolamine N-methyltransferase (PEMT), which converts phosphatidylethanolamine (PE) to PC via three sequential methylation reactions. This pathway may partially buffer low choline intake but does not fully compensate for a systemic choline deficit. A common functional single-nucleotide polymorphism in the PEMT gene (rs7946, G523A) results in a Valine to Methionine substitution at position 175 (V175M), which reduces PEMT activity (92) and this has been associated with an increased risk of sporadic AD in a Chinese population for APOE ε4 non-carriers and female subjects (92, 93).

#### 3.2.6 Vitamin B5 (pantothenic acid)

Vitamin B5, also known as Pantothenic acid or ‘anti-stress vitamin’, is an obligatory precursor of coenzyme A (CoA), known to play an essential role in different metabolic processes such as fatty acid metabolism, synthesis of neurotransmitter and biogenic pathways like TCA cycle (94). Moreover, CoA contributes to the function and structure of the brain, thanks to its role the synthesis of fatty acids, cholesterol, amino acids, phospholipids and steroid hormones (69). In 2020, a study by Xu and colleagues showed different levels of vitamin B5 between AD cases and controls in different brain regions, discovering that the quantity of the vitamin was lower in AD to 30-50% of control values. The difference was observed in the hippocampus, entorhinal cortex, and middle temporal gyrus, with high vitamin B5 levels mostly colocalizing with white matter. Observations in postmortem tissue do not explain if vitamin B5 depletion is a consequence or cause of neuropathological process, but the authors suggest vitamin B5 deficiency may be an early perturbation in AD. Low levels of vitamin B5 could affect the production of CoA and acetyl-CoA and its uptake in Krebs cycle with negative consequence on the cerebral urea metabolism. The authors conclude that neurodegeneration associated with vitamin B5 deficiency may be preventable with an oral supplementation (95).

#### 3.2.7 Vitamins B6 (pyridoxine, pyridoxal, and pyridoxamine)

Vitamin B6 vitamers (pyridoxine, pyridoxal, and pyridoxamine) are converted to pyridoxal 5’-phosphate, an essential coenzyme for various biochemical reactions. The difference in efficacy of these vitamers is suggested by Oppici et al., which reports that pyridoxamine and pyridoxal form are more active compared to pyridoxine in the context of pharmacological treatment for patients with hyperoxaluria type I (96). Vitamin B6 is essential for the correct function of the nervous system for many reasons. It is involved in regulation of oxidative stress and takes part in neurotransmitter production. The vitamin is an important coenzyme in the synthesis of the following neurotransmitters: norepinephrine (NE), noradrenaline, glutamate, DA, 5-HT and GABA (69, 71, 97, 98). Moreover, pyridoxine is involved in the conversion of homocysteine to cysteine (99). High homocysteine levels are associated with an increased risk for AD (100). An upregulation in vitamin B6 catabolism in AD patients compared to controls was reported (101).

#### 3.2.8 Vitamin B7 (biotin)

Vitamin B7, known also as Biotin or Vitamin H, has a key role as a cofactor of enzymes that are involved in glycogen synthesis, amino acids and fatty acids metabolism. It is also required for the histone biotinylating process, an epigenetic modification involved in cell proliferation, gene silencing and DNA damage repair. Its importance in gene expression is underscored by the fact that it can affect the expression of more than 2000 genes in human cells (102–104). Vitamin B7 contributes to the nervous system’s well-functioning not only for the regulation of important genes for neuronal health, but also because this vitamin helps in oxidative stress reduction in neurons and supports mitochondrial activity (105). Although biotin was not deeply studied in the AD field, its importance in the nervous system suggests that it can help to protect neurons, potentially reducing the risk of neurodegenerative events (106).

#### 3.2.9 Vitamin B8 (inositols)

Vitamin B8 indicates inositols, sugar-like compounds that the human body can synthesize. The supply of inositol in the brain comes from (1) Phosfatidyl-Inositols derivative recycling, (2) active transport of inositol from the periphery and (3) *de novo* synthesis. The inositol role in the brain attracted attention because inositol levels in the cerebrospinal fluid (CSF) are 7-fold higher than plasma levels, and in some brain regions they are also found to be 50- to 200-fold higher (107, 108). Inositols have multiple stereoisomers, which are present in both intra- and extracellular compartments of neuronal and glial cells. Epi- and scyllo-inositol isomers can stabilize the non-toxic forms of Aβ, they have antioxidant capacity (107, 108) and they can act as second messengers of the insulin-signaling pathway. Due to their insulin-mimetic and insulin-sensitizing properties, they are able to increase insulin sensitivity, with glucose level reduction in the blood and promotion of hepatic glycogen synthesis (109).

#### 3.2.10 Vitamin B9 (folic acid)

Vitamin B9, also known as folic acid, is required for basic processes like cell repair, DNA synthesis, methylation and homocysteine regulation (110). In the context of the brain, it is important to note its crucial role in maintaining synaptic plasticity, which is necessary for a proper memory function (111). This vitamin is also necessary during neurodevelopment and its decrease is associated with defects of the neural tube and a reduction in ACh synthesis (111). It is required for supporting mitochondrial health and it is involved in the process of oxidative stress reduction (113). Folates are essential for life, not only because of their major role in essential biochemical reactions, like the metabolism of nucleotides, but also for DNA methylation, which in turn is a significant mechanism for epigenetic transmission, responsible for gametogenesis and embryo development. Dna methylation, together with histone methylation, regulates in a very specific way the expression of pivotal genes in imprinting (112). Vitamin B9 is required for the synthesis of catecholamines (DA, NE, epinephrine) and 5-HT, which are important neurotransmitters for the function of brain areas such as the cerebral cortex, limbic system, and hypothalamus. Moreover, it has been discovered that its deficiency can increase Aβ plaque (113). This vitamin deficiency is more frequently observed in older individuals and is linked not only to neuronal damage, but also to cognitive decline and memory problems (114) indicating a risk factor for AD (115). In a recent NHANES-based cross-sectional study of 2,104 adults ≥60 years, higher total dietary folate, folic acid intake, and red blood cell (RBC) folate levels were associated with lower odds of global cognitive impairment, with RBC folate showing the strongest linear inverse association (OR 0.86, 95% CI 0.75–0.97 per unit increase). However, when stratified by CVD status, the protective effect of higher RBC folate on cognition was significantly attenuated in participants with CVD compared to those without CVD. Overall, the study suggests that adequate folate status supports global cognitive function in older adults, but coexisting CVD may blunt this benefit (116).

#### 3.2.11 Vitamin B10 (PABA)

Para-aminobenzoic acid (p-aminobenzoic acid, PABA or 4-aminobenzoic acid) is often classified as vitamin B10 or BX. PABA is not synthesized in mammals but is a fundamental component of metabolism and can be easily absorbed by the intestine due to the supply of food and symbiotic bacteria which produce it constantly (117). PABA is an irreplaceable substrate for folic acid synthesis and it is produced through folic acid breakdown (118). Moreover, it has been identified as Coenzyme Q precursor (119), and crucial for DNA synthesis and replication (120). Vitamin B10 is known for its anti-cancer, anti-AD, antibacterial, antioxidant, antiviral, and anti-inflammatory properties, making PABA compounds potential therapeutic agents for future clinical trials (121). PABA-building block is already used as a scaffold for many drugs (121, 122) and recently for the development of a potential cholinesterase inhibitor for AD treatment (123). See section 4.2.10.

#### 3.2.12 Vitamin B12 (cobalamin)

Vitamin B12 is required for the nervous system preservation, during development and in adult life, being involved in cognitive functions such as concentration and memory (124). It is known to be important for homocysteine metabolism, DNA synthesis and repair, a mechanism that is fundamental for proper function of post-mitotic cells like neurons. Vitamin B12 is also known for its protective role thanks to its antioxidant effect, as it indirectly participates in glutathione and H₂S biosynthesis (125). Evidence from *in vitro* studies, in melanocytes, showed that Vitamin B12 can induce the activation of Nuclear factor erythroid 2-related factor 2 (NRF2) triggering in this way transcription of catalase (CAT) and superoxide dismutase (SOD) genes that are responsible for the ROS scavenging. Vitamin B12 is able to induce SOD and CAT upregulation also in neuronal cells, as demonstrated by Theiss and co-workers in SH-SY5Y model (126). In addition, Vitamin B12 is required for myelin formation, regulation of neurotransmitters, synthesis of the catecholamines (71). Moreover, this vitamin can help to recover the integrity of the blood–brain barrier (BBB), impaired in AD (127). As a cofactor of methionine-synthase, vitamin B12 is important for the homocysteine conversion into methionine. Its subclinical deficiency leads to an intracellular increase of homocysteine (128), which in turn is able to increase oxidative stress and inflammation (129). Vitamin B12 deficiency is associated with defects in myelin production, nerve damage, neurodegenerative symptoms, cognitive decline, dementia and memory deficits (130). In a longitudinal study with 7 years of follow up, the involvement of homocysteine and holotranscobalamin (holoTC) were suggested to be involved in AD development (129).

#### 3.2.13 Vitamin B13 (orotic acid)

The orotic acid, known as non-canonical vitamin B13, has an essential role in the *de novo* synthesis of pyrimidine, the building blocks of DNA and RNA. A continuous supply of pyrimidine nucleotides is important for the proper CNS development, regeneration, plasticity and neurotransmission in the adult brain (131). It is known that some P2Y receptor subtypes are sensitive to uridine nucleotides. The P2Y receptors belong to the G-protein-coupled receptors (GPCRs) that can be activated after binding of extracellular nucleotides (such as ATP, ADP, UTP and UDP), triggering physiological brain processes such as neurotransmission, regulation of synaptic functions or release of synaptic vesicles. It is not surprising that all three genes of the pyrimidine *de novo* synthesis pathway are expressed in many rat brain regions such as the hippocampus, neocortex and cerebellar cortex (132, 133). Moreover, AD patients have altered mRNA levels of genes involved in pyrimidine synthesis (134). Orotate has also anti-inflammatory activity, through uridine-monophosphate formation, inhibits cellular apoptosis and promotes cellular growth (135). Moreover, vitamin B13 has been recently used as a ‘stabilizing’ agent for metal ions and absorption. Indeed, metal ion imbalance in AD can disrupt organelles, impair mitochondria and autophagy, and promote Aβ and tau aggregation (136) and restoring metal homeostasis could improve AD outcomes. Finally, the discovery of its antioxidative effects in improvement of membrane electrical conductivity and inhibition of free radical formation, suggests its possible benefit as a neuroprotective agent (137).

#### 3.2.14 Vitamin B15 (pangamic acid)

Vitamin B15, discovered in 1938, is also known as pangamic acid, pangametin and sopangamine (138), it occurs naturally, and it has been used for decades as a cellular respiration stimulator. In addition to the natural form, a synthetic product known as di-isopropyl-ammonium dichloroacetate (DIPA) is available with comparable biological properties (139). In Germany, there is a commercially available vitamin B15 drug, although pangamic acid refers to three chemically distinct compounds: gluconic acid, glycine and diisopropylammonium dichloroacetate. Moreover, both dimethylglycine hydrochloride (DMG) or diisopropylamine dichloroacetate (DIPA-DCA) are sold as vitamin B15 on the market (140). In addition, dichloroacetate, as part of diisopropylamine dichloroacetate, is demonstrated to be harmful to both human and animal as mutagenic (141, 142). Given the enormous confusion regarding this vitamin identity, and the fact that no reliable scientific evidence supports its classification or efficacy, vitamin B15 remains a controversial and ill-defined compound in biomedical literature (143). The Food and Drug Administration identifies vitamin B15 as “not an identifiable substance” (140). In addition, no associations with AD have emerged from 1938 to date.

#### 3.2.15 Vitamin B17 (amygdalin or laetrile)

Vitamin B17, also known as amygdalin, is a cyanogenic glycoside chemical that can be found in kernels and fruit pulp. It is also available as a synthetic compound named laetrile (142). No associations with AD have emerged to date.

#### 3.2.16 Vitamin C

Vitamin C is a water-soluble compound with antioxidant properties and is necessary for many biosynthetic processes, such as those of collagen synthesis and carnitine metabolism (144) and it is a micronutrient necessary for neurodevelopment and neurotransmission, as well as for maintaining the redox balance (145). The balance maintenance also occurs through iron, zinc, and copper chelation (19). Evidence suggests that vitamin C is involved in neurotransmission through aminergic (DA, NE and 5-HT), glutamatergic, and cholinergic mechanisms. Specifically, it is implicated in the synthesis of catecholamines through the modulation of hydroxylase enzymes, particularly tyrosine hydroxylase. The transcription of this enzyme is increased by vitamin C, which is also involved in the synthesis of L-DOPA (146). Additionally, vitamin C is required for the conversion of DA into NE through the reaction catalyzed by the enzyme dopamine-β-hydroxylase and can inhibit glutamate cytotoxicity through an inhibitory effect on NMDA receptors and by reducing glutamate-mediated AMPK receptor phosphorylation (145).

Vitamin C reaches the CSF via sodium-dependent vitamin C transporter type 2 (SVCT2) or through GLUT transporters from the blood and it has been shown that systemic vitamin C deprivation leads to a reduction in hippocampal volume (10-15%), impairing learning and memory abilities (146). In addition, lower vitamin C levels were associated with increased cognitive deficits in AD patients, who showed reduced plasma vitamin C concentrations than healthy patients. This link, bearing in mind the multiple activities of vitamin C in neuroprotection, suggests its fundamental role in the AD onset and progression (145). Dixit and colleagues confirmed this correlation in their study published in 2015. They generated a novel mouse model of vitamin C deficiency in AD by crossing SVCT2 heterozygous knockout mice with a bigenic mouse carrying two mutations known to cause early-onset AD (SVCT2+/−APP/PSEN1+). These mice have intracellular vitamin C deficiency, but normal circulating levels. They showed that mice with low vitamin C had an increased lipid peroxidation and a decrease in antioxidant potential, accelerating Aβ production and deposition and the progression of the associated cognitive deficits (147).

Regarding the protective role of vitamin C on the formation of Aβ plaques, its mechanism of action has not yet been known, although it seems clear that oxidative stress plays a fundamental role (145). However, a study by Sampaio et al. proposed a biochemical and mechanistic explanation of the neuroprotective process performing a Molecular dynamics (MD) experiment. Briefly, ascorbic acid appears to have a strong affinity for the region between the D23 residue and the K28 residue, competing with Aβ molecules in the formation of oligomers and destabilizing pre-formed ones (148).

In summary, Vitamin C plays an important role in the synthesis of neurotransmitters as well as ROS scavenger. There are associations between reduced cognitive abilities and low vitamin C levels in AD patients.

## 4 Vitamin supplementation in AD patients and preclinical models

The use of vitamins as a treatment for neurodegenerative diseases has been explored by several research groups and an excellent systematic review of studies conducted between 2011 and 2021 was written by Martinez and colleagues (16). A more recent review describes the importance of vitamins in neurodegenerative diseases (14). In the present review, we focused on studies specifically aimed at studying AD in human, animal or *in vitro* studies. In this way, we provide readers with an overall view of the state of the art for each vitamin.

### 4.1 Fat-soluble vitamins

#### 4.1.1 Vitamin A

Although there are not enough clinical trials to define the effects of vitamin A supplementation on the progression of AD, several experimentations have been performed on animal models. Investigation on APP/PS1 mouse models have demonstrated the ability of vitamin A dietary supplementation to inhibit Aβ aggregation. Specifically, the supplementation of 15 IU/g vitamin A for 12-week showed decreased Aβ deposition and better learning and memory abilities (although not significant) (149). These data were confirmed by another study where Vitamin A supplementation in VAD shows the same effect in memory (150) and demonstrate the ability of vitamin A to protect and assist the regeneration of neurons during neurodegenerative process (151). The effect of vitamin A supplementation in AD has been further demonstrated in a study on APP/PS1 mice treated with intraperitoneally administered RA for 8 weeks. The authors showed a robust decrease in Aβ brain deposition and pTau, reduced activation of microglia and astrocytes, attenuated neuronal degeneration, and improvement of spatial learning and memory compared to control animals (151).

It would seem that gender is a determining factor for the progression of the disease and for the response to treatment with vitamin A (20 IU/g). In 3xTg-AD transgenic mice, it was observed that the accumulation of Aβ was 10 times higher in females than males. After treatment with vitamin A, Aβ40 and Aβ42 levels were decreased in male mice, while they were increased in female mice. Regarding the pTau, the quantity was found to be equal in both male and female mice, but differences were observed in the response to vitamin A treatment. In male mice the supplementation decreased the amount of pTau due to a reduction in CDK5 levels, a kinase necessary for the phosphorylation of the tau. This did not occur in female mice. The authors explain that, during menopause, the ovarian estrogen production decreases progressively and that this production is regulated by RA. In fact, when supplemented with vitamin A, female mice activate a compensatory mechanism that transports vitamin A to the ovaries instead of the hippocampus. However, in the ovaries there is an enzyme that inactivates estrogen, regulated by RA. This activates a vicious circle by further decreasing circulating estrogen levels and increasing vitamin A seizure from the brains of mice (152).

In summary, vitamin A seems to exert neuro-regenerative and anti-aggregative effect in male mice. In menopausal mice, vitamin A is hijacked to the ovaries, favouring the Aβ accumulation in the brain.

#### 4.1.2 Vitamin D

In recent years, several animal and clinical studies have been conducted to determine whether vitamin D (in the active form of D3) supplementation may play a role in AD progression. Research by Morello in 2018 showed that feeding 5XFAD transgenic mice with a high vitamin D3 intake (7,500 IU/kg for 4 months), significantly improves working memory when administered during the early stages of the disease. Conversely, in later stage, high vitamin D supplementation fails to exert protective effect on cell proliferation and neurogenesis (153). These effects on Wistar rats have also been confirmed by Mehri et al. in 2020. They showed that Aβ-treated mice that received vitamin D (5 μg/kg/day for 2 weeks) had an increase in spatial and learning memory, similar to the control groups. This improvement has been linked to vitamin antioxidant effect with a reduction in lipid peroxidation and DNA damage along with a decrease in total antioxidant capacity (TAC) and total thiol groups (TTG) (as an indicator of GSH/GSSG ratio) in the hippocampus and serum samples of the same animals (154). These results are in line with the evidence of Yu and colleagues who observed an improved cognitive function accompanied by a decrease in Aβ load, along with an increase in NGF levels and a reduction in inflammatory markers in the brains of treated AβPP-PS1 transgenic mice (12 IU/g vitamin D for 3-4 weeks). Moreover, authors show an alteration in Amyloid Precursor Protein (APP) expression with a variation in APP end products depending on vitamin D supplementation (155). Thus, from a biochemical and molecular point of view, all these therapeutic effects can be explained by considering the interaction of vitamin D on its receptors at the CNS level, which triggers neuronal protection against the degenerative processes of AD, including anti-inflammatory action, anti-atrophic effect by regulating neurotrophic agents as NGF and GDNF, attenuating the accumulation of the Aβ42 peptide by stimulating its phagocytosis and increasing the efflux through the BBB (156, 157). Animal studies are consistent with the few available clinical studies on Vitamin D-supplemented AD patients. Among these, a trial of patients with AD who received 800 IU/day vitamin D revealed improved cognitive function and a significant decline trend in Aβ-related biomarkers, such as APP and BACE1, after 12 months of treatment (158) (Table 1).

**Table 1:** Vitamin D-based clinical trials.

Treatment	Populations	Main results	Reference and/or NCT number
100,000 IU/4 weeks Vitamin D3 for 12 weeks (study protocol) Placebo	Moderate AD patients with vitamin D deficiency	Not Available (N. A.)	NCT01409694 (241)
800 IU/day Vitamin D (*n* = 105) for 12 months Placebo (*n* = 105)	AD patients	Improvement in cognitive function and decrease in Aβ-related biomarkers in elderly patients with AD.	ChiCTR-IIR-16009549 (158)
50,000 IU/week vitamin D3 for 8 weeks (*n* = 10) Placebo (*n* = 9)	AD patients with vitamin D deficiency	Increase in plasma Aβ, particularly in older adults, suggesting decrease in brain Aβ.	(242)
800 IU/day Vitamin D (*n* = 93) for 12 months Placebo (*n* = 90)	MCI population	Improved cognitive function, reduction in oxidative stress, increase in telomere length.	(243)
1,000 IU/day vitamin D2 (low-dose) for 8 weeks and then high-dose D (based on serum 25OHD, 6,000 IU/day) for 8 weeks (*n* = 16) 1,000 IU/day vitamin D2 (low-dose) for 8 weeks and then placebo for 8 weeks (*n* = 15)	Mild or moderate AD patients	High-dose vitamin D (6,000 IU/day) provides no benefit for cognition or disability over low-dose vitamin D in mild–moderate AD patients.	(244)

Along with the benefits of vitamin D, it is also important to consider the work of Lai and colleagues, according to which “Vitamin D supplementation worsens Alzheimer’s progression” in animal model and in Aβ42-exposed SH-SY5Y cells. Authors tried to give a molecular explanation to their result by investigating the activation of the VDR pathway following the interaction with vitamin D, finding that the vitamin D did not rescue the canonical VDR pathway but instead further activated the VDR/p53 complex causing damage to AD brains. In addition, they performed retrospective studies that confirmed their conclusion in AD subjects with advanced stage disease (159). However, these results have been extensively commented on and discussed by Gombart et al. highlighting the limitations of the study by Lai and colleagues in terms of approach and methodology (37). To date, the precise mechanism and activity are still debated, and more studies will be necessary to acquire this knowledge.

In Table 1, the available clinical trials based on vitamin D supplementation are reported.

In summary, vitamin D treatment seems to be effective in early stage of the disease, impacting Aβ clearance and leading to oxidative stress and Aβ-markers reduction. Animal experiment reported also a reduction in neuro-inflammation and lipid peroxidation.

#### 4.1.3 Vitamin E

Studies on mice have shown that vitamin E dietary supplementation is inversely correlated with the incidence of AD. Sung et al., investigating the effect of vitamin E supplementation in transgenic APP mice (Tg2576), highlighted that vitamin E can lead to a reduction in Aβ aggregation in young individuals that received a diet supplemented with 2 IU/g vitamin E, but not in older ones when Aβ plaques were already deposited (160). A study on double mutant mice [Ttpa(-/-)APPsw] obtained by crossing knockout mice [Ttpa(-/-)], with high levels of cerebral oxidative stress, with APP transgenic mice (APPsw) showed that plasma Aβ40 and Aβ42 levels were reduced in mice receiving the α-tocopherol-supplemented diet (750 mg of α-tocopherol/kg) from weaning to the sacrifice (53, 161).

To emphasize the vitamin E protective effects, it is necessary to mention the study conducted by Nasri et al., who evaluated the effects of supplementation with Tocotrienol Rich Fraction (TRF), on APPswe/PS1dE9 double transgenic mice, an AD mouse model. After 6 months of treatment, an upregulation in Slc24a2 gene expression, which plays a neuroprotective role and maintains neuronal plasticity, was observed. In support of this, knockout mice for this gene showed deficits in motor learning and spatial working memory in the hippocampus. On the other hand, Pla2g4a gene, which was downregulated by the treatment, is associated with the APP protein expression, which induces the aggregation of Aβ in the cortical region (162).

In addition to its effect on Aβ plaques, vitamin E exerted a protective action also on pTau. APP transgenic mice that overexpressed tau protein at brain level, following a diet with 800 IU/Kg of α-tocopherol acetate showed a decrease in pTau and improved cognitive performance. Different kinases are responsible for this hyperphosphorylation including p38 (p-p38). The results show that a high level of p-p38 protein is present in the hippocampus of transgenic mice, whereas this is not observed in the control group; moreover, this difference is not present in the mice cortex. When supplemented with vitamin E in the diet, p-p38 levels returned to control values (163).

Regarding the effect on oxidative stress, an interesting evaluation was conducted on APOE-deficient mice with a diet supplemented with 1% of α-tocopherol. After a 12-months treatment, the supplemented mice showed better behavioral performance compared to those that received a normal diet. The lipid peroxide and glutathione levels remained within normal limits (164). A second study on 3xTg-AD transgenic mice highlighted that the diet supplemented with α-tocopherol mitigated the increase in the levels of GSSG, and lipid peroxidation in both the cortex and hippocampus. Also in this case an improvement of cognitive function has been detected (165).

Although animal studies have shown that vitamin E has beneficial effects on AD pathogenesis, its effectiveness in humans is not clear. Some studies have shown benefits when vitamin E was given to AD patients [such as the study by Dysken et al. (167, 168) (Table 2)], while others have claimed the opposite. Specifically, Lloret et al. observed that some AD patients treated with 800 IU/day of vitamin E for 6 months were “non-responsive.” In these patients, oxidative stress did not decrease after treatment and cognitive function decreased more than in placebo-treated patients (Table 2) (169). In this case, the authors have hypothesized that vitamin E acted as a pro-oxidant molecule. In this context, it should be also noted that excessive vitamin E supplement increases all-cause mortality, as reported by Miller et al. (170).

**Table 2:** Vitamin E-based clinical trials.

Treatment	Populations	Main results	Reference and/or NCT number
2000 IU/day alpha-tocopherol and placebo (*n* = 152) for 6 months to 4 years, Placebo and 20 mg/day of memantine (*n* = 155), 2000 IU/day alpha-tocopherol and 20 mg/day of memantine (*n* = 154), Placebo (*n* = 152).	Diagnosis of possible or probable AD patients	Slower functional decline in α-tocopherol-treated patients.	NCT00235716 (167, 168)
Placebo and a multivitamin daily (*n* = 240). Vitamin E (2000 IU) and placebo and a multivitamin daily (*n* = 240) Placebo and Donepezil (10 mg) and a multivitamin daily (*n* = 240)	MCI patients	N.A.	NCT00000173 (245)
2000 IU/day Vitamin E and multivitamin once a day for 3 years (*n* = not found) Placebo and Multivitamin once a day (*n* = not found)	Down syndrome with concomitant AD patients	N.A.	NCT00056329
2000 IU/day Vitamin E for 3 years (*n* = not found) Placebo (*n* = not found)	Down syndrome with concomitant AD patients	N.A.	NCT01594346
1 g fish oil, 22 mg carotenoids and 15 mg vitamin E daily for 12 months (*n* = 50) Placebo (*n* = 27)	Mild or moderate AD	Improved memory and mood.	(246)
800 IU/day vitamin E (*n* = 19) for 6 months Placebo (*n* = 14)	AD patients	At the end of the study, the authors found two groups of patients: respondents, where the vitamin E showed a therapeutic effect, and non-respondents, where it was ineffective. In the respondent group with vitamin E, GSSG levels were lower and the cognitive status was maintained. In the “non-respondent” group, vitamin E was not effective in preventing oxidative stress, and cognition decreased sharply.	(169)
2000 IU/day vitamin E (*n* = 20) for 6 months 10 mg/day donepezil (*n* = 20)	Mild to moderately severe probable AD patients	Worsening neuropsychological test scores in vitamin E-treated patients.	(247)
400 mg/day Vitamin E in for 6 months (*n* = not found)	AD patients	Decreased levels of oxidative stress markers.	(56, 166)
2000 IU/day vitamin E for 3 years (*n* = 257) 10 mg donepezil daily (*n* = 253) Placebo (*n* = 259)	AD patients	No significant differences in the rate of progression to AD between the vitamin E and placebo groups at any time-point.	(250)
2000 IU/day Vitamin E (*n* = 15 mild, *n* = 15 moderate to severe AD) for 6 months Donepezil 10 mg/day (*n* = 15 mild, *n* = 15 moderate to severe AD)	Mild, moderate or severe AD patients	Improved neuropsychological tests.	(249)

In Table 2, the available clinical trials based on vitamin E supplementation are reported.

In summary, clinical trials are contrastant reguarding Vitamin E treatment. It is likely that only some people (“responding population”) may benefit from treatment with the vitamin E.

#### 4.1.4 Vitamin K

Although a clear correlation between serum and cerebral vitamin K levels with progression of AD and cognitive decline has been described, to date, no clinical trials have been reported in the scientific literature on the effect of vitamin K supplementation on AD. Many reviews report that the pleiotropic effects of vitamin K, from the neuroinflammation and oxidative stress regulation to the impact on comorbidities in the elderly (osteoporosis and CVD), and this could justify a clinical trial in AD patients (171, 172).

Several *in vitro* studies have been carried out to elucidate the impact of vitamin K supplementation on neurodegeneration. Hadipour et al. showed that treating PC12 cells with vitamin K2 (5–200 μM) reduces neuronal death caused by Aβ42 or hydrogen peroxide (H_2_O_2_) treatment. This treatment decreased the level of apoptotic signaling proteins and ROS amount, and increased glutathione concentration. The antioxidant and protective activity were linked to the anti-inflammatory effect of vitamin K through the p38 MAPK pathway inactivation (173). This result is perfectly in line with the study by Huang and colleagues in rat astroglia C6 cell lines transfected with APP. They showed that, in addition to the antioxidant activity, vitamin K2 could also protect nerve cells from Aβ toxicity through activation of the PI3K/Akt/Bad signaling pathway and inhibition of caspase-3 mediated apoptosis. Furthermore, the Gas6/Axl receptor, one of the main modulators of cell survival and proliferation, as well as the myelination process (174), appears to be the targets of PI3K/Akt/Bad signaling in AD (175).

As confirmation, an AD transgenic *Drosophila* model was treated with vitamin K2 for 28 days and an improved climbing ability, prolonged lifespan and decreased Aβ42 levels have been observed. Moreover, LC3 and Beclin1 upregulated expression and decreased p62 level, as autophagy markers in AD flies, have been detected. These findings suggested that vitamin K2 can activate autophagy pathways contributing to the Aβ brain clearance, thus reducing its neurotoxicity (176). In addition, vitamin K2 has been shown to increase ATP levels and reduce cognitive decline in isoflurane-exposed FAD5X transgenic mice that express Aβ in the brain. After vitamin K treatment (100 mg/kg/day), they had more synapses and higher levels of ATP in their hypothalamus, resulting in cognitive improvement (177). Therefore, although existing studies highlight the therapeutic potential of vitamin K supplements, further *in vivo* trials are needed to confirm its efficacy and fully understand its therapeutic relevance from a molecular and clinical point of view.

In summary, no clinical trial were conducted on vitamin K in AD field, although it is neuroprotective in preclinical model, favouring the autophagy and Aβ clearance in the brain and counteracting the oxidative stress.

### 4.2 Water-soluble vitamins

#### 4.2.1 Vitamin B1 (thiamine)

Data supporting the link between vitamin B1 deficiency and the pathophysiology of AD attracts interest in the possible supplementation of this vitamin with therapeutic intent. A study targeting an older adult population examined the association between vitamin B1 dietary intake and cognitive decline. The results of the examination show a significant association between a higher dietary vitamin B1 intake with higher processing speed, higher executive function scores and better cognitive performance (76). Another study, published by Gibson and colleagues, was performed with a daily 600 mg supplement of Benfotiamine, the synthetic fat-soluble form of vitamin B1, designed to enhance bioavailability and cellular uptake. Researchers evaluated the clinical decline primarily by AD Assessment Scale-Cognitive Subscale (ADAS-Cog) and secondarily with clinical dementia rating (CDR) score and fluorodeoxyglucose (FDG) uptake. The results from the study show a 43% non-significant increase of ADAS-Cog in the treated group, suggesting a possible reduction in cognitive decline. Moreover, the treatment was able to induce a significant 77% lower worsening in CDR, with a stronger effect in APOE4 non-carriers. Finally, FDG pattern score showed a significant effect after 1 year of treatment. Although these results seem promising and indicate that oral administration of Benfotiamine is safe, the study is limited by its small sample size (34 patients in the benfotiamine group and 36 in the placebo group) and short duration (12 months) (178).

In Table 3, the available clinical trials based on vitamin B1 supplementation are reported.

**Table 3:** Vitamin B1-based clinical trials.

Treatment	Populations	Main results	Referenceand/or NCT number
600 mg/day Benfothiamine for 12 months (*n* = 34) Placebo (*n* = 36)	Amnestic MCI or mild dementia due to AD	Less cognitive decline. Significant reduction of advanced glycation end products (AGE) in blood.	NCT02292238 (178)
600 mg/day Benfothiamine for 72 weeks 1200 mg mg/day Benfothiamine Placebo (study protocol)	Early AD patients	N.A.	NCT06223360 (250)
3 g/day thiamine hydrochloride for 3 months (*n* = not found) Placebo	AD patients	Cognitive improvement	(251)
100 mg/day Fursultiamine (*n* = 8) for 12 weeks	AD patients	Emotional, intellectual and other mental functions improvement.	(252)
3 g/day Thiamine for 1 year (*n* = not found) Placebo	AD patients with dementia	No significant differences between the placebo and thiamine groups at any point during the study.	(253)
Sulbutiamine (400 mg/day the first month, and then 600 mg/day) and Donepezil (5 mg/day the first month, and then 10 mg/day) (*n* = 40) for 6 months Placebo and Donepezil (*n* = 43)	Early AD patients	Improved daylife activities scores in both groups in mild AD patients.	(254)

In summary, Thiamine is tested in clinical trials, recording cognitive improvement in AD populations.

#### 4.2.2 Vitamin B2 (riboflavin)

Vitamin B2 intake has been associated with better cognitive performance. Regarding its mechanism of action, one main hypothesis is that the vitamin increases cognitive performance by reducing oxidative stress through its antioxidant properties (179, 180). A cross-sectional study with 2,893 participants was published recently. The aim of the research was to explore the association between vitamin B2 intake and cognitive performance by using different assessment tests. The vitamin B2 intake was calculated as the means of two 24 h-dietary recall interviews. The results from the study showed a significant association between vitamin B2 intake and the cognitive scores which measure the ability to immediately recall information as soon as it is presented (Odds Ratio = 0.77) if compared to participants with a Vitamin B2 intake below the recommended dietary allowance. A higher vitamin B2 intake was negatively associated with the risk of low cognitive performance. Moreover, the researchers reported that physical activity may modify the link between Vitamin B2 and cognitive performance (181). Another study published in 2023 by Zhou with older adults has been performed. Vitamin B2 intake was classified again as the previous study by data coming from two 24 h-period interviews and cognitive evaluation has been performed. The results indicate that higher intake of vitamin B2 is associated with higher score on each of the used cognitive tests. Interestingly if the group with higher vitamin B2 intake is compared to the last quartile there is significant 45.1-fold increase in DSST test scores (*p* = 0.004). Further research is needed to assess the causal link between increased consumption of food with high vitamin B2 content and cognitive performance (182).

In summary, Vitamin B2 is not currently used in clinical trials, but dietary interviews point out the direct correlation between this vitamin intake and cognitive score.

#### 4.2.3 Vitamin B3 (niacin)

Recent evidence shows the possible use of vitamin B3 in AD treatment. In a study 44 participants were randomly assigned to 40 mg of statins (lovastatin / simvastatin / pravastatin sodium) per day treatment or 1 g of extended-release niacin per day, for 6 weeks. The authors measured the 24S-hydroxycholesterol (24S-HC) blood levels since recent evidence shows that the serum levels are higher in AD patients (183). 24S-HC represents the major product of cholesterol metabolism in the brain, and about 80 % of the 24S-HC can be found there. Its levels may be considered a marker for neuronal death because it is known to cause release of cholesterol from cellular membranes. Moreover, it is demonstrated that brain cholesterol is converted to 24S-HC, transferred across the BBB and eliminated as bile acids (184, 185). In the cited study, authors showed that extended-release niacin was able to significantly reduce the 24S-HC serum level by 10% and LDL cholesterol by 18.1% without lowering the plasma concentration of APOE. Also, statin treatments were able to reduce 24S-HC serum level, in a higher percentage. In conclusion, authors propose this class of drugs as beneficial for AD treatment due to their direct effect on brain cholesterol metabolism. Future studies are needed to understand the mechanism by which 24S-HC levels are reduced, and to assess if this treatment can influence other AD markers as Aβ plaques (183, 186). Unfortunately, beyond positive effects on cholesterol metabolism, another clinical trial (187) found no improvements in AD-related markers in CSF or in cognitive function after the administration of 3000 mg/day of nicotinamide (ammidic form of niacin) for 48 weeks to 41 patients (MCI and AD with positive AD biomarkers).

In summary, vitamin B3 seems to impact the cholesterol metabolism, but further studies on classic markers of AD have to be conducted to confirm a recent study.

#### 4.2.4 Vitamin B4 (choline)

Choline seems to exert a preventive effect on AD onset. In this respect, Poly and collaborators correlated choline dietary intake and cognitive function in adult human subjects (188). As corollary, treatment with certain choline-containing compounds showed a limited tendency in reducing cognitive impairments in human vascular dementias in small clinical trials (188, 189). Probably, dietary choline intake may influence cognitive function through the effect of PC-containing eicosapentaenoic and docosahexaenoic acids, whose levels are known to be reduced in brains from AD patients (190). Morena and collaborators demonstrated an improvement in cognition and global function in the AD population treated with choline alfoscerate after 3 and 6 months (191).

Also, a study published by Dave and coworkers suggests the importance of choline intake by diet to reduce AD hallmarks. In the study, 3xTg-AD mice that underwent a choline-deficient diet from the age of 3 to 12 months, showed a greater deficit with hippocampal network and postsynaptic membrane dysregulation, as well as inflammation and altered mitochondrial dysfunction (192).

Choline was also associated with a neuroprotective role. High choline intake in the perinatal periods protects from neural dysfunction, including that coming from aging in many animal models (193–195). Intriguingly, choline proved to be an interesting transgenerational supplement. The researchers tested the transgenerational beneficial effects of choline maternal supplementation (5 g/kg choline chloride) for two consecutive generations of AβPP-PS1 mice. The results showed that the supplement was able to induce changes in genes related to inflammation, neuronal death, histone modifications, and a decrease of brain homocysteine level in the hippocampus with the final effect of AD pathology reduction (196).

In Table 4, the few available clinical trials based on vitamin B4 supplementation are reported.

**Table 4:** Vitamin B4-based clinical trials.

Treatment	Populations	Main results	Reference and/or NCT number
1200 mg/day Choline alfoscerate (*n* = 132) for 6 months Placebo (*n* = 129)	Mild to moderate AD	Improvement in cognition and global function	(191)
Choline alfoscerate and Donepezil (*n* = not found) Placebo and Donepezil alone (*n* = not found)	AD patients with cerebrovascular injury	N.A.	NCT02648906
2.2 g/day choline bitartrate for 6 months (*n* = not found)	Pre-symptomatic AD (people with increased risk of AD)	N.A.	NCT05880849
1200 mg Choline alfoscerate and Donepezil 5 mg or 10 mg daily for 48 weeks (recruiting) Placebo and Donepezil 5 mg or 10 mg alone,	Diagnosed as a probable AD patients	N. A.	NCT05383183
400 mg Choline alfoscerate and Donepezil 10 mg daily for 24 weeks (*n* = not found) Donepezil 10 mg, (*n* = not found)	AD patients with dementia or probably AD patients	N. A.	NCT03441516
1200 mg Choline alfoscerate and donepezil (*n* = 50) daily for 24 weeks Donepezil alone (*n* = 50)	AD patients	Choline alfoscerate exhibits improvement in both cognitive and non-cognitive domains	(255)

In summary, there are preliminary studies in human and animal models which highlight the potential use of vitamin B4 in the AD field.

#### 4.2.5 Vitamin B5 (pantothenic acid)

Despite the importance of this vitamin in both functional and structural roles in the brain, and data suggesting its possible correlation to AD (95), to our knowledge, no clinical, *in vivo* or *in vitro* studies have been conducted on supplementation of this vitamin in AD context so far.

#### 4.2.6 Vitamins B6 (pyridoxine, pyridoxal, and pyridoxamine)

Calderon-Ospina et al., in 2020 showed that daily supplementation with this vitamin for longer than 6 months in a dosage >500 mg/day had neurological reversible side effects. This toxic dose can lead to necrosis, axon degeneration and damage to nerve cell bodies; however, interruption of the supplementation allows nerve regeneration (197). In a study published by Choi et al., the authors evaluated the effect of membrane-free stem cell extract and pyridoxal 5-phosphate treatment in Aβ_25-35_-injected AD mice. They showed that this combination significantly downregulated the expression of amyloidogenic-related proteins improving learning capabilities (198). This vitamin has frequently been used in combination with other B vitamins as discussed in detail in the dedicated section below.

#### 4.2.7 Vitamin B7 (biotin)

Although, to our knowledge, a direct link between vitamin B7 and AD is missing, its role in neuronal function and energy metabolism suggests a possible protective effect. Its antioxidant capacity and mitochondrial support may protect neurons from dysfunction (102, 199).

#### 4.2.8 Vitamin B8 (inositols)

Although relatively few studies have explored the role of inositols in AD, emerging hypotheses involving brain insulin resistance and lipid dysregulation are fueling interest in these compounds as potential therapeutic agents. Among these, D-pinitol and related inositol isomers have gained attention for their insulin-sensitizing and neuroprotective properties, with potential to improve brain insulin signaling and mitigate neurodegeneration (200).

Vitamin B8 has been tested in various clinical and preclinical models as a potential AD therapy. For example, scyllo-inositol was evaluated in a clinical trial involving 353 participants, completed by 139 participants (placebo and treatment groups) over 78 weeks, at doses of 250, 1000, or 2000 mg/day. Although the 250 mg group showed a non-significant increase in brain ventricular volume, CSF levels of Aβ42 were significantly reduced (Table 5) (201). *In vitro* findings by McLaurin and colleagues further support the relevance of inositol stereoisomers in AD. These compounds stabilize Aβ oligomers and inhibit their toxicity by forming non-toxic complexes in PC-12 cells and primary human neuronal cultures (202). Conversely, allo-inositol exhibited only a weak inhibitory activity against Aβ toxic species in biochemical assays (203). In AD models, Pitt and colleagues demonstrated that mature hippocampal neurons responded to pinitol (3-O-methyl DCI), glycan INS-2 and D-chiro-inositol (DCI) with increased phosphorylation of upstream insulin signaling components. Notably, DCI significantly enhanced insulin’s neuroprotective capacity against Aβ-induced synaptic degeneration, preserving neuronal integrity (204). More recently, Medina-Vera et al. assessed the effects of chronic D-pinitol administration (200 mg/kg/day for 18 weeks) in the 5xFAD mouse model of AD. The treatment improved cognitive performance, reduced hippocampal Aβ deposition, increased expression of insulin-degrading enzyme (IDE), a key enzyme in Aβ clearance, elevated circulating ghrelin and leptin levels, and restored PI3K/Akt insulin signaling (205).

**Table 5:** Vitamin B8-based clinical trials.

Treatment	Populations	Main results	Reference and/or NCT number
250 mg/day Scyllo-inositol (*n* = 52) for 78 weeks 1000 mg (*n* = 15) 2000 mg (*n* = 19) Placebo (*n* = 53)	Mild to moderate AD patients	Aβ42 was decreased significantly in CSF	NCT00568776 (201)
250 mg/day Scyllo-inositol for 12 weeks (*n* = 157) Placebo (*n* = 157)	AD patients with symptoms of agitation and aggression	N.A.	NCT01735630
250 mg/day Scyllo-inositol for further 36 weeks to both the previous arms (extension study)	Patients who completed NCT01735630 study	Plasma SAM and SAM/SAH levels were significantly higher in treated patients, while Aβ40, PS1-mRNA, and TNF-α-mRNA levels were lower.	NCT01766336
6 mg/day Inositol for 4 weeks (*n* = 12 cross-over placebo trial)	AD patients	No significant differences	(256)

Given their ability to cross the BBB (206, 207) and interfere with Aβ aggregation, specific inositol isomers represent promising candidates for AD therapy. Ongoing and completed clinical trials investigating vitamin B8 supplementation are summarized in Table 5.

In summary, inositols are a class of interesting candidates able to impact both the insulin resistance and the Aβ deposition in the brain.

#### 4.2.9 Vitamin B9 (folic acid)

A trial by Chen and co-workers supported the idea that folic acid supplements might mitigate AD by reducing inflammation. All AD participants (donepezil as basic routine therapy) were randomly assigned to the control group or intervention group (1.25 mg/day folic acid) for 6 months. The intervention was able to induce a significant decrease of both TNF-α mRNA and protein, serum decrease of Aβ40 (but not the Aβ42), and a decrease of PS1-mRNA. The study concluded that folic acid enhanced cognition and reduced inflammatory markers, though donepezil’s presence may have influenced the findings (Table 6) (208). Nevertheless, the study was for a short duration and the optimal dose of folic acid needed to be improved (209). Another trial published by Ma et al. in 2015, detected the effects of folic acid supplementation on cognitive function in adults with mild cognitive impairments (MCI) (159 participants). Folic acid was administered at 400 μg/day for 6 months by the oral route. Analysis of biomarkers and cognitive performance were performed at 3 and 6 months. At 6 months, the supplement improved significantly the Full-scale IQ, Digit Span, and Block Design. The authors concluded that a longer duration in the administration was desirable (210) and the same authors performed a second study with a longer treatment period (12 months) of folic acid supplementation evaluating the cognitive performance. Patients with MCI were assigned to the active group folic acid 400 μg/day or placebo; the treatment gave significant improvement in folate, homocysteine, Aβ42, peripheral IL-6 and TNF-α concentrations. Moreover, the supplement significantly improved cognitive performance and reduced inflammatory cytokine levels in the periphery (211). For studies in combination with other B vitamins, see the dedicated section below.

**Table 6:** Vitamin B9-based clinical trials.

Treatment	Populations	Main results	Reference and/or NCT number
1.25 mg/day Vitamin B9 + donepezil for 6 months (*n* = 61) Donepezil (*n* = 60)	Newly diagnosed patients with mild to severe AD	Plasma SAM and SAM/SAH levels were significantly higher, while Aβ40, PS1-mRNA, and TNF-α-mRNA levels were lower in the intervention group than in the control group.	ChiCTR-TRC-13003246 (208)
400 μg/day vitamin B9 for 6 months (*n* = 80) Conventional treatment (*n* = 79)	MCI	Full-scale IQ, Digit Span, and Block Design improved	(210)
400 μg/day vitamin B9 for 12 months (*n* = 77) Conventional treatment (*n* = 75)	MCI	Full-scale IQ, Digit Span, and Information improved. Decrease in peripheral inflammation and Aβ42.	(211)
1 mg/day vitamin B9 and cholinesterase inhibitors (*n* = 23) for 6 months Placebo and cholinesterase inhibitors alone (*n* = 18)	Probable AD patients	Improvement of instrumental activities of daily living and social behavior scores	(257)

In Table 6, the available clinical trials based on vitamin B9 supplementation are reported.

In summary, vitamin B9 was able to reduce inflammation and Aβ-linked markers in clinical trials.

#### 4.2.10 Vitamin B10 (PABA)

PABA derivatives have been used recently for the development of a potential cholinesterase inhibitor for AD treatment. In particular, Shrivastava and coworkers synthesized a series of PABA derivatives and evaluated them *in vitro*. They showed that the major part of the compounds had inhibitory effects against acetylcholinesterase (AChE) and butyrylcholinesterase (BChE) enzymes. *In vivo* experiments in 4-5 months old albino strain rats (Charles Foster rats) with these compounds exhibited significant reversal of cognitive deficits as amnesia, compared to treatment with the standard drug donepezil. Finally, authors confirmed that PABA derivatives had AChE inhibition activity on specific brain regions such as hypothalamus, hippocampus and prefrontal cortex (123). To our knowledge, no clinical trial have been conducted on supplementation of this vitamin in AD context so far.

#### 4.2.11 Vitamin B12 (cobalamin)

Rosenberg and Miller were among the first to propose that subtle vitamin deficiencies could contribute to neurocognitive decline in aging (212). They suggested that incorrect function of OCM may play a crucial role in AD etiology and in this context the increase of total homocysteine might be the marker of such dysregulation (115, 213). The hypothesis has led to increased focus on the B vitamins involved in homocysteine metabolism, especially vitamin B12. A trial by Dangour et al. in 2015 assessed the effect of Vitamin B12 supplementation in the context of cognitive function. Participants had moderate vitamin B12 deficiency (107–210 pmol/L), and no significant cognitive impairment. Participants from the active group take 1 mg of vitamin B12 daily for 12 months. The results from this study pointed out that there is no beneficial effect on cognitive and neurological function for patients who take the supplement (214). Many studies involving vitamin B12 have been conducted in combination with other B-complex vitamins, yielding more pronounced beneficial effects; these findings are discussed in greater detail in the dedicated section below.

#### 4.2.12 Vitamin B13 (Orotic acid)

To our knowledge vitamin B13 was used only in combination with vitamins such as vitamin B12 and D3, or glycosides (see dedicated section below).

#### 4.2.13 Vitamin B15 (Pangamic acid)

Vitamin B15 has presented several problems in the classification of compounds indicated under this name. To our knowledge, there are no studies that associate its deficiency or supplementation with AD.

#### 4.2.14 Vitamin B17 (amygdalin or laetrile)

To date, only one study has reported a potential direct link between vitamin B17 and AD. In 2024, Kalaimathi and colleagues conducted an *in silico* analysis in which amygdalin, along with other phytochemicals such as eriocitrin, keracyanin, and amaroswerin, was identified as a potential natural inhibitor of BACE1. These compounds exhibited superior predicted absorption and distribution profiles compared to the synthetic BACE1 inhibitor CNP520, and, notably, did not present the hepatotoxic effects associated with the latter. While these computational findings support the therapeutic relevance of plant-derived molecules in modulating amyloidogenic processes, their efficacy and safety must still be confirmed through *in vivo* experimental validation (215). We did not find additional studies directly linking vitamin B17 to AD, but other researchers suggest it may play a role in supporting neuronal health. She et al., reported a neuroprotective effect of amygdalin together with two other glycosides (paeoniflorin and astragaloside IV) in a rat model of cerebral ischemia. In particular, 0.128 g/kg dose of glycosides showed significant effect in the improvement of neurological dysfunction, neuronal damage alleviation, and neuronal pyroptosis inhibition. The study also investigates molecular mechanisms that underline this effect, finding that the 0.128 g/kg group has a significant inhibition of the NLRP3, caspase-1, pro-caspase-1, ASC, and IL-1β expression, which are proteins of the NLRP3-induced pyroptosis (216). In a recent second paper published in 2025 by Kimura and coworkers, the amygdalin’s possible protective role in acute ischemic stroke model has been examined. Vitamin B17 was administered intraperitoneally at different doses (5 mg/kg, 10 mg/kg and 20 mg/kg) 24 h post-reperfusion for a duration of 3 consecutive days. The results showed that amygdalin significantly reduced the cerebral infarct volume induced by occluding the right middle cerebral artery for 30 min. Moreover, amygdalin was able to decrease caspase-9 expression and uncleaved caspase-3 expression, leading the authors to conclude that it plays a neuroprotective role modulating the apoptosis process (217).

In summary, vitamin B17 is not currently used in clinical trials, although it seems like interesting candidate to stimulate a neuroprotection through the inhibition of pyroptosis and BACE-1.

#### 4.2.15 Vitamin C

Several studies provided evidence to support the therapeutic role of vitamin C in AD. In a rat model treated with trimethyltin chloride (TMT) to stimulate neurodegenerative damage, vitamin C alone or combined with aerobic exercise showed positive results. Rats receiving 4 mg/Kg vitamin C showed Nrf2 and SOD levels in the hippocampus comparable to those of the control group, reversing the decrease induced by TMT. PI3K and CAT levels were higher in the group treated with both vitamin C and aerobic exercise compared to the TMT-treated group (218).

An interesting finding was highlighted by experiments conducted on specific AD mouse models that are unable to synthesize vitamin C on their own (KO-Tg AD). High-dose vitamin C supplementation in these mice reduced Aβ plaque formation in the cortex and hippocampus by decreasing BBB damage and restoring normal mitochondrial activity (219). An analysis conducted on 1953 patients demonstrated that, although BBB permeability increases with age, it is higher in AD patients compared to healthy individuals. This alteration leads to the typical AD vascular issues (220). An interesting result obtained on KO-Tg mouse models has shown that supplementation with vitamin C at a concentration of 3.3 g/L not only reduced the amount of Aβ in the hippocampus and cortex but also decreased the BBB destruction (219). These results are further supported by an *in vitro* study on SH-SY5Y Aβ-exposed cells showed that vitamin C pretreatment prevented apoptosis as well as cell death, in addition to decreasing basal rates of generation of endogenous Aβ (221).

Finally, in the AβPP-PSEN1 mice model, Harrison and colleagues have shown that parenteral injections of ascorbic acid improved cognitive abilities, despite not improving either oxidative stress levels or Aβ aggregation (222). On the other hand, orally administered vitamin C reduced ROS and proinflammatory cytokines induced by Aβ peptide injections in the hippocampus in rat brains (223).

In summary, vitamin C is not currently used in clinical trial, although there is evidence in preclinical models showing that AD patients could benefit from a reduction in BBB destruction.

## 5 Vitamin cocktails in AD patients and preclinical models

Up to this point, we have reviewed the dietary importance of vitamins and highlighted their various biological effects at the basis of their therapeutic potential. On the other hand, we are aware that in multifactorial pathologies such as AD, it is quite unlikely that the administration of a single vitamin can balance the complex clinical feature of the pathology. For this reason, this last section discusses the effects of combined vitamin administration, identifying perspectives and limitations of these studies.

Regarding the lipophilic vitamins given in combination, only a few studies have been carried out. Starting from animal experimentation, we must mention the study by Mehrabadi et al., which compared the effects of vitamin D3 (1 μg/kg) and vitamin E (100 mg/kg) supplementation (alone and in combination) on AD Wistar rats (obtained through intra-hippocampal injection of Aβ40). They showed that a long-term vitamins E + D3 combined administration inhibits oxidative stress by modulating levels of malondialdehyde (MDA) and SOD, in a more efficient way than when these vitamins are given alone. Moreover, they observed a delay in the progression of the pathology and aging of rats, highlighting a decrease in neuronal loss and morphological changes (224).

On the other hand, Joshi and colleagues focused on the benefits of Vitamin A and E combination in Aβ aggregation, both *in vitro* and *in vivo* on *C. elegans*. Specifically, *in vitro* aggregation tests were carried out with a mixture of RA (0-7 molar equivalents (ME)) and α-tocopherol (0.15-0.25 ME). Surprisingly, while these two molecules alone were able to inhibit the Aβ aggregation *in vitro*, the combination wasn’t effective. As mentioned above (Paragraph 3.1.3), aggregated forms of α-tocopherol are able to promote the Aβ aggregation, while monomeric forms inhibit it. In the co-presence of vitamin A, even monomeric forms lost their protective effect, making the co-treated sample perfectly comparable in terms of Aβ aggregation to the untreated one. However, the effect was different on the *C.elegans* AD model, where a decrease in Aβ aggregates was observed in RA and α-tocopherol co-treated worms, with a corresponding increase in their mobility after treatment (28).

So far, there are no studies that describe the combinatory use of vitamins A + D in AD patients. Noteworthy, the A + D3 combination was studied on lymphoid cells. Vitamins D3 and A have similar receptor types. Specifically, the nuclear VDR creates homodimers with retinoid X receptor (RXR) and can bind RA, or it heterodimerizes with RXR and binds to vitamin D responsive elements (VDREs). That means that vitamin D and A can compete for receptor binding, neutralizing each other’s effects (225). Indeed, it is also observed that an increase in vitamin A intake and high serum retinol levels are linked to an increased risk of bone fracture and frailty, confirming this competitive mechanism (1, 226).

The effects of vitamin E (200 mg) supplementation, in combination with vitamin C (300 mg), and β-carotene at various concentration (16.7, 8.4, 5.6, or 0 mg/day), compared with those of vitamin E alone, were evidenced in a study conducted on 276 elderly subjects aged between 60 and 75 years. Comparing plasma Aβ levels, a reduction (although not significant) was observed in all treatment groups compared to the beginning of the trial. However, patients treated with vitamin E + C showed lower Aβ plasma levels and improved cognitive abilities compared to vitamin E alone-treated ones (227). This result is perfectly in line with another prospectic study. The combined supplementation of vitamin C + E (whether co-administered with other multivitamins or not) had a strong negative correlation with the AD incidence. The supplementation of vitamin E alone with multivitamins had the same trend on AD incidence but with a smaller magnitude than the vitamin combination (228).

Hydrophilic B vitamins attracted considerable interest, as evidenced by numerous clinical studies that have tested their use in different combinations and timings. In particular, vitamin B9 is often used in combination with B6 and B12, since they are required for homocysteine conversion into methionine (129). The efficacy of B vitamin supplementation remains debated, with outcomes differing across studies. Among the clinical trials that failed to demonstrate cognitive benefits from B vitamin supplementation, one by Eussen et al. (229) reported no significant improvement after 6 months of treatment with vitamin B12 (1 mg/day) and B9 (0.4 mg/day). Similarly, a longer 18-month trial by Aisen et al. (230) on 340 individuals with mild-to-moderate AD showed that, although high-dose supplementation of B9 (5 mg/day), B6 (25 mg/day), and B12 (1 mg/day) effectively reduced homocysteine levels by 31%, it failed to slow cognitive decline (Table 7). Conversely, several studies have reported beneficial effects of B vitamin supplementation. For instance, a randomized controlled trial by Soininen et al. demonstrated that a 2-year intervention with Souvenaid (Fortasyn Connect), a multinutrient formulation containing B6, B9, B12, as well as choline (B4), and vitamins C, E, and selenium, led to improved memory performance and slowed cognitive decline in patients with prodromal AD. While B vitamins support homocysteine metabolism, a synergistic effect with antioxidant components such as C, E, and selenium may also contribute to these positive outcomes (231). Another study enrolled 133 individuals with MCI older than 70 years, who received either placebo or a daily combination of B9 (0.8 mg), B12 (0.5 mg), and B6 (20 mg) for 2 years. Notably, those with elevated baseline homocysteine levels (≥11.3 μmol/L) experienced a 50% reduction in brain atrophy rate and improvements in episodic memory, semantic memory, and overall cognition (232–234). A follow-up study on the same cohort found that the neuroprotective effect of B vitamin supplementation was observed only in participants with high plasma omega-3 fatty acid levels (>590 μmol/L). These findings suggest a synergistic interaction, where the efficacy of omega-3 fatty acids in reducing brain atrophy may depend on adequate B vitamin status (235). In another trial involving 299 men over 75 years old, a 2-year supplementation with folate (2 mg), vitamin B6 (25 mg), and B12 (400 μg) significantly reduced plasma Aβ40 levels, suggesting a potential role of B vitamins in modulating amyloid metabolism (236). Finally, a study in AD patients receiving vitamin B9 (1.2 mg/day) and B12 (50 μg/day) for 6 months reported significant increases in plasma S-adenosylmethionine (SAM) and SAM/SAH ratio, along with reductions in serum homocysteine, plasma SAH, and TNF-α levels, highlighting the therapeutic potential of this approach (209).

**Table 7:** Vitamin cocktails administered in clinical trials.

Vitamins	Treatment	Populations	Main results	Reference and/or NCT number
Vitamin D and B9	1600 IU vitamin D3 and 400 pg vitamin B9 (*n* = 133) daily for 24 weeks 400 pg vitamin B9 only (*n* = 134) Placebo (*n* = 135)	Early stage of AD patients with MCI	Co-treatment did not improve cognitive function	(258)
Vitamin E and C	800 IU Vitamin E, 200 mg vitamin C, and 600 mg α-lipoic acid daily for 4 months (*n* = 24) 800 mg Coenzyme Q daily (*n* = 20) Placebo (*n* = 18)	Patients with mild to moderate AD	Aβ42, tau, and P-tau181 levels did not change in treated patients. CSF isoprostane levels, an oxidative stress biomarker, decreased on average by 19% from baseline to week 16 in the treated group but were unchanged in the other groups.	NCT00117403 (259)
Vitamin E and C	400 IU vitamin E and 1000 mg vitamin C, daily for 1 month (*n* = 10) 400 IU vitamin E alone (*n* = 10)	Clinically probable AD patients	vitamin E + C supplementation decreased lipid peroxidation level, while vitamin E alone was not effective.	(260)
Vitamin B1, B6, B9 and B12	50 mg vitamin B1, 50 mg vitamin B6, 5 mg vitamin B9, and 0.05 mg vitamin B12 daily for 3 months (*n* = 27) No treatment (*n* = 25)	Patients with AD and MCI	Reduced levels of carbonyl proteins	(261)
Vitamin B9, B12	Vitamin B12 (1 mg/day) and B9 (0.4 mg/day) for 6 months (*n* = 51) Placebo (*n* = 57) Vitamin B12 (1 mg/day) (*n* = 54)	Older people with mild vitamin B-12 deficiency	Any improvement in cognitive function	(229)
Vitamin B6, B9, B12 (commercial supplement)	Vitamin B9 (5 mg/day), B12 (0.4 mg/day), and B6 (50 mg/day) for 12 months (*n* = 78) Placebo (*n* = 67)	Patients with MCI	No improvement in quality of life, cognitive function wasn’t evaluated	(262)
Vitamin B6, B9, B12	25 mg Vitamin B6, 5 mg vitamin B9 and 1 mg vitamin B12 daily for 18 months (*n* = 202) Placebo (*n* = 138)	Patients with AD and mild or moderate cognitive impairment.	The vitamin supplement regimen was effective in reducing homocysteine levels, but it had no beneficial effect on the primary cognitive measure	NCT00056225 (230)
Vitamins B6, B9, B12, B4, C, E, and other ingredients (fortasyn connect, commercial supplement)	1 mg vitamin B6, 0.4 mg vitamin B9, 3 μg vitamin B12, 0.4 mg vitamin B4, 40 mg vitamin E, 80 mg vitamin C, and Selenium (60 μg), EPA (300 mg), DHA (1200 mg), phospholipids (106 mg), UMP (625 mg) for 2 years (*n* = 120) Placebo (*n* = 125)	Prodromal AD	Slowed cognitive decline	(231)
Vitamin B6, B9, B12	0.8 mg Vitamin B9, 20 mg vitamin B6, 0.5 mg vitamin B12 daily for 24 months (*n* = 85) Placebo (*n* = 83)	Patients with MCI	In subjects with high baseline serum ω-3 fatty acids, B vitamin treatment slowed the mean atrophy rate in the brain. B vitamin treatment had no significant effect on the rate of atrophy among subjects with low baseline serum ω-3 fatty acids.	ISRCTN94410159 (VITACOG study) (232–234, 235)
Vitamin B6, B9 and B12	2 mg Vitamin B9, 25 mg vitamin B6 and 400 μg vitamin B12 for 24 months (*n* = 125) Placebo (*n* = 130)	AD patients	B vitamins decreased the Aβ plasma level.	(236)
Vitamin B9, B12	1 mg of methylcobalamin and 5 mg of folic acid daily for 24 months (*n* = not found) Placebo (*n* = not found)	Patients with AD or vascular dementia	Improvement in cognitive performance and behavioral and psychological symptoms of dementia in treated patients.	NCT00164970
Vitamin B9, B12 and E	Tablet containing 400 μg Vitamin B9, 6 μg Vitamin B12, 30 IU α-tocopherol (vitamin E); 400 mg SAM, 600 mg N-acetyl cysteine and 500 mg Acetyl-L-carnitine taken daily for 3, 6, 12 months (*n* = 49) Placebo (*n* = 34)	Patients with clinical diagnosis of AD or MCI	Improved cognitive performance and mood/behavior in 1 year-treated patients.	NCT01320527 (264, 265)
Vitamin B9, B12	1.2 mg/day Vitamin B9 and 50 μg/day vitamin B12 for 6 months (*n* = 60) Placebo group (*n* = 60)	Patients clinically diagnosed as probable AD and in stable condition	Supplementation significantly increased plasma SAM and SAM/SAH, and significantly decreased the levels of serum Hcy, plasma SAH and serum TNF-α.	(209)

All cocktails are intended custom, except those indicated as “commercial supplement.”

Interestingly, vitamin B9 + E combination was evaluated on an AD mice model. More specifically, Aβ-injected Swiss mice were treated with B9 (25 mg/Kg) + E (250 mg/Kg), or with B9 (50 mg/Kg) + E (500 mg/Kg) for 14 days. An improvement in spatial memory and a reduction in synaptic dysfunction and in the expression of neuronal (nNOS) and inducible (iNOS) nitric oxide synthase forms was observed. This effect is mainly due to the presence of vitamin E that converts NO• to nitrite ester, which is less toxic to neurons, causing a decrease in neuronal loss (237). Moreover, the higher dosage restored the mitochondrial activity of complexes I, II, and IV. On the other hand, these treatments were ineffective on glial cell inflammation, suggesting that the protective effect is independent of anti-inflammatory features (238). Thus, further studies are mandatory to better understand the mechanisms by which the combination of folic acid and α-tocopherol acts to prevent neurodegeneration for the rational design of new therapeutic approaches.

As mentioned earlier in Section 4.2.12, vitamin B13 has not been studied alone, but only in combination with other vitamins, as demonstrated in two studies of major interest in the AD context. In both papers, vitamin B13 was administered as magnesium orotate and although the authors’ attention has focused mainly on magnesium, the relevance of orotic acid cannot be excluded. In the first one, Viel and co-workers reported the use of a vitamin cocktail in the TgF344-AD rat model. The cocktail was composed by magnesium orotate (500 mg/kg) + benfotiamine (300 mg/kg) + vitamin B9 (10 mg/kg) + B12 (1 mg/kg) + D3 (75 μg/kg) (B13 + B1 + B9 + B12 + D) and was administered daily for 14 days by oral gavage. The researchers reported that reduced mitochondrial respiration was fully restored in cocktail treated-transgenic rats, while the wild-type rats did not respond to this treatment. Moreover, it facilitates the release of hippocampal ACh during physiological stimulation. Authors suggested that the supplement therapy may be possibly beneficial for cholinergic and mitochondrial function in AD (239). In the second paper, the authors showed that different combinations of folic acid, magnesium orotate, and vitamin B6 have significant effects on glycolysis, Aβ production and aggregation in SH-SY5Y-APP_695_ cells and *C. elegans*. They showed that the combination of magnesium orotate and vitamin B6 significantly reduced Aβ40 levels in SH-SY5Y-APP_695_ cells. Authors concluded that there are both advantages and disadvantages in single or combined administration, but the combination leads to synergic effects and enhances positive outcomes (240).

In Table 7, a summary of the available clinical trials based on vitamin cocktail supplementation is reported.

In summary, Vitamin C and E supplementation reduces the risk of AD onset and provides greater protection than vitamins alone. Vitamins B6, B9 and B12 are often used in combination, but to achieve tangible results, they must be administered for at least 2 years. The Fortasyn Connect formulation contains all five of the above vitamins and other ingredients that has achieved excellent results. Additional formulations such as A + E, A + D, B9 + E, B13 + B1 + B9 + B12 + D have demonstrated interesting effects at the preclinical level, and further studies are desirable.

## 6 Conclusion

Nutrition represents a form of prevention and personal care that not only impacts physical appearance and well-being in the short term but is likely to slow down disease onset such as neurodegenerative diseases. In this review we have presented evidence regarding the close link between hypovitaminosis and AD pathology. These may be due to dietary problems, either related to malnutrition and intestinal diseases or to poor dietary education (such as the Western diet). As thorough as we have tried to be in describing all the various known hypovitaminosis, we have not considered combinations of hypovitaminoses. Doing this would require more population studies, which are unfortunately scarce to date.

We have also evaluated the effects that a supplement of specific vitamins may produce in AD-related biological systems. In our opinion, the studies reported in this review are not the solution to the AD problem, but in their own way they provide pieces of a tremendously complex puzzle. In this area, we see two great limitations: on the one hand, a critical limitation in the use of vitamins for AD is that no single vitamin can adequately target the numerous contributing factors to its pathogenesis, and on the other hand, the short duration of many current studies prevents a comprehensive evaluation of their therapeutic impact.

Finally, we evaluated the effects of vitamin combinations as prevention and treatment of AD. Indeed, in modern medicine it is known that superior efficacy is often achieved by combining bioactive molecules in appropriate ratios rather than solely increasing their concentration. Unfortunately, at the present moment there are very few studies, and more effort is needed in this respect, especially with regard to dose-finding of certain combinations. In fact, it seems clear that the OCM (managed by vitamins B6 + B9 + B12) is crucial for AD and targeting it is one of the best strategy so far. Future research should be designed to replicate the conditions under which positive effects were observed, with a particular focus on long-term interventions. Notably, trials reporting improvements in cognitive impairment were typically those lasting 2 years. Given the reduced neuroplasticity in the brains of AD patients compared to younger individuals, it is reasonable to assume that longer treatment durations may be required to achieve meaningful therapeutic effects. Not surprisingly, the best treatment effects were also seen in the early stages of the disease.

Another important aspect that we believe is worth noting is the fact that in some of the studies presented, researchers identified populations that responded to treatment and those that did not. This complicates and broadens the picture, but on the other hand, it may require researchers to find new factors to consider in the exclusion and inclusion criteria for clinical trials.

Finally, we must not forget that AD is not a dysmetabolism that can be solved by administering the missing metabolite; it is a disease where we have to keep track of everything that is not working, and we have to reconstruct a parafunctional pathway. This is precisely why a balance of all these factors and metabolites that are missed in the AD patient is necessary. The work presented in this review is intended as a support for future clinical trials in the treatment of AD.
