# Supplementary material for: Vitamin deficiencies and Alzheimer’s disease: evidence and implications for supplementation
Source: Front Nutr. 2026 Feb 13;13:1676497. doi: 10.3389/fnut.2026.1676497 (PMC12947704; doi:10.3389/fnut.2026.1676497)
Supplement: Supplementary file 1 [file Table_1.docx]

***Supplementary Material***

**Table S1**. A summary of the known activities of lipophilic vitamins in the nervous system and Population Reference Intakes (PRI)/ Adequate Intake (AI) according to EFSA [1], Reference Daily Intakes (RDIs) according to Office of Dietary Supplements (ODS) of National Institutes of Health (NIH) [2]

| **Vitamin** | **PRI / AI (EFSA)** | **RDIs**  **(ODS-NIH)** | Known activities in the nervous system | **Reference** |
| --- | --- | --- | --- | --- |
| **A**  **Pubchem:**  [**445354**](https://pubchem.ncbi.nlm.nih.gov/compound/445354) | **PRI:**  **♂ : 750 µg of retinol equivalent (RE)/day**  **♀ : 650 RE/day** | **RDIs:**  **♂♀ : 900 µg RE/day** | ● Involved in nerve cell proliferation and differentiation.  ● Involved in Ca^2+^ dependent signaling, by controlling both synaptic plasticity and neuronal firing  ● Synthesis and release of neurotransmitters  ● Protective role against Aβ plaques formation (by stimulating α-secretase enzyme), tau hyperphosphorylation and degradation. | [3], [4], [5], [6] |
| **D**  **Pubchem:**  [**483926502**](https://pubchem.ncbi.nlm.nih.gov/substance/483926502) | **AI:**  **♂♀ : 15 µg/day** | **RDIs:**  **♂♀ : 20 µg/day** | ● Involved in nerve cell proliferation and differentiation.  ● Synthesis of neurotransmitters, including acetylcholine (ACh), γ-aminobutyric acid (GABA), dopamine (DA), and 5-HydroxyTryptamine (5-HT), and neurotrophic factors  ● Protective role against Aβ plaques formation, tau hyperphosphorylation and their degradation. | [7], [8], [9], [10] |
| **E**  **Pubchem:**  [**483926503**](https://pubchem.ncbi.nlm.nih.gov/substance/483926503) | **AI:**  **♂ : 13 mg/day** α**-tocoferol**  **♀ : 11 mg/day** | **RDIs:**  **♂♀ : 15 mg/day** α**-tocoferol** | ● Antioxidant activity  ● Protective role against Aβ plaques formation and degradation  ● Involved in nerve cell proliferation and differentiation.  ● Involved in neurotransmission | [11], [12], [13] |
| **K**  **Pubchem:** [**5280483**](https://pubchem.ncbi.nlm.nih.gov/compound/5280483) | **EFSA (AI)**  **♂♀ : 70 µg/day Phylloquinone (K1)** | **RDIs:**  **♂♀ : 120 µg/day** | ● Involved in nerve cell proliferation and differentiation  ● Involved in sphingolipids synthesis in both neuronal and glial cell membranes  ● Responsible for the inhibition of ferroptosis | [14], [15], [16] |

**Table S2**. A summary of the known activities of hydrophilic vitamins in the nervous system and Population Reference Intakes (PRI) / Adequate Intake (AI) according to EFSA, Reference Daily Intakes (RDIs) according to Office of Dietary Supplements (ODS) of National Institutes of Health (NIH)

| **Vitamin** | **PRI / AI (EFSA)** | **RDIs**  **(ODS-NIH)** | Known activities in the nervous system | **Reference** |
| --- | --- | --- | --- | --- |
| **B1 (thiamine)**  **Pubchem:** [**1130**](https://pubchem.ncbi.nlm.nih.gov/compound/1130) | **PRI:**  **♂♀ : 0.1 mg/MJ** | **RDIs:**  **♂♀ : 1.2 mg/day** | ● Essential for oxidative energy metabolism  ● Involved in the synthesis and function of neurotransmitters, including ACh, GABA and glutamate  ● Involved in synapses formation and myelin synthesis | [17], [18], [19] |
| **B2 (riboflavin)**  **Pubchem:** [**493570**](https://pubchem.ncbi.nlm.nih.gov/compound/493570) | **PRI:**  **♂♀ : 1.6 mg/day** | **RDIs:**  **♂♀ : 1.3 mg/day** | ● Substrate for the synthesis of flavin mononucleotide (FMN), and flavin adenine dinucleotide, used in energy and lipid metabolism, thyroid hormone regulation, iron utilization and glutathione redox cycle  ● Involved in myelin synthesis | [20], [21] |
| **B3 / PP (niacin)**  **Pubchem:** [**936**](https://pubchem.ncbi.nlm.nih.gov/compound/936) | **PRI:**  **♂♀ : 1.6 mg Niacin Equivalent (NE) NE/MJ** | **RDIs:**  **♂♀ : 16 mg NE/day** | ● Substrate for the synthesis of nicotinamide adenine dinucleotide (NAD+) and nicotinamide adenine dinucleotide phosphate (NADP+) nucleotides.  ● Antioxidant activity  ● Key mediator of neuronal development and survival | [22] |
| **B4 (choline)**  **Pubchem: 305**  In some countries and according to Pubchem, vitamin B4 corresponds to adenine.  **Pubchem:** [**190**](https://pubchem.ncbi.nlm.nih.gov/compound/190) | **AI:**  **♂♀ : 400 mg/day** | **RDIs:**  **♂♀ : 550 mg/day** | ● Involved in the synthesis of phosphatidylcholines  ● Precursor of the ACh neurotransmitter  ● Involved in One-carbon metabolism (OCM) | [23], [24] |
| **B5 (pantothenic acid)**  **Pubchem:** [**6613**](https://pubchem.ncbi.nlm.nih.gov/compound/6613) | **AI:**  **♂♀ : 5 mg/day** | **RDIs:**  **♂♀ : 5 mg/day** | ● Substrate for the synthesis of coenzyme A (CoA)  ● Involved in the synthesis of neurotransmitters, such as ACh  ● Involved in the synthesis of steroid hormones, cholesterol, phospholipids, fatty acids and amino acids | [19], [20] |
| **B6**  **Pubchem:**  [**104817**](https://pubchem.ncbi.nlm.nih.gov/compound/104817)  According to Pubchem, B6 vitamins corresponds to :  pyridoxine  **Pubchem:** [**1054**](https://pubchem.ncbi.nlm.nih.gov/compound/1054)  pyridoxal  **Pubchem:**  [**1050**](https://pubchem.ncbi.nlm.nih.gov/compound/1050)  pyridoxamine  **Pubchem:**  [**1052**](https://pubchem.ncbi.nlm.nih.gov/compound/1052) | **PRI:**  **♂ : 1.7 mg/day**  **♀ : 1.6 mg/day** | **RDIs:**  **♂♀ : 1.7 mg/day** | ● Involved in the synthesis of neurotransmitters, including DA, 5-HT, GABA, noradrenaline, glutamate and of the hormone melatonin  ● Involved in lipid metabolism  ● Involved in the breakdown of homocysteine | [17], [19], [20], [25], [26], [27], [28] |
| **B7 / H (biotin)**  **Pubchem:** [**171548**](https://pubchem.ncbi.nlm.nih.gov/compound/171548) | **AI:**  **♂♀ : 40 µg/day** | **RDIs:**  **♂♀ : 30 µg/day** | ● Involved in gluconeogenesis, fatty acid synthesis and amino acid metabolism (biotin-dependent carboxylases)  ● Involved in gene expression (histone biotinylation) of over 2000 genes, including insulin receptor and human thiamine-transporter-2 | [19], [29], [30] |
| **B8 (inositol)**  **Pubchem:** [**892**](https://pubchem.ncbi.nlm.nih.gov/compound/892)  In some countries and for Pubchem, vitamin B8 correspond to Adenosine monophosphate  **Pubchem:** [**6083**](https://pubchem.ncbi.nlm.nih.gov/compound/6083) | **Undetermined RDA** | **Undetermined RDIs** | ● Involved in insulin signaling pathway (insulin-mimetic and insulin-sensitizing molecule)  ● Antioxidant activity  ● Substrate in the formation of important derivatives in neuronal excitability involved in the GABAergic and glutamatergic signaling | [31] |
| **B9 (folic acid)**  **Pubchem:** [**135398658**](https://pubchem.ncbi.nlm.nih.gov/compound/135398658) | **PRI:**  **♂♀ : 330 µg Dietary Folate Equivalents (DFE) DFE/day** | **RDIs:**  **♂♀ : 400 µg DFE/day** | ● Involved in the synthesis of nucleic acid and amino acid metabolism  ● Involved in the breakdown of homocysteine  ● Involved in methylation process, maintaining neuronal and glial membrane lipids  ● Involved in synthesis of neurotransmitters including 5-HT and catecholamines | [19], [26], [27], [28] |
| **B10 ( p-aminobenzoic acid (PABA))**  **Pubchem:** [**978**](https://pubchem.ncbi.nlm.nih.gov/compound/978) | **Undetermined RDA** | **Undetermined RDIs** | ● Substrate in the synthesis of folic acid by the gut microbiota  ● Antioxidant activity | [32] |
| **B12 (cobalamin)**  **Pubchem:** [**5311498**](https://pubchem.ncbi.nlm.nih.gov/compound/5311498) | **AI:**  **♂♀ : 4 µg/day** | **RDIs:**  **♂♀ : 2.4 µg/day** | ● Involved in the metabolism of homocysteine  ● Involved in neural myelination and synaptogenesis  ● Stimulates neural survival and resilience  ● Antioxidant and anti-inflammatory activity  ● Involved in Kennedy pathway (homocysteine recycle)  ● Involved in the de novo DNA synthesis and methylation | [17], [26], [27], [28], [33], [34], [35] |
| **B13 (orotic acid)**  **Pubchem:** [**967**](https://pubchem.ncbi.nlm.nih.gov/compound/967) | **Undetermined RDA** | **Undetermined RDIs** | ● Involved in the synthesis of pyrimidine nucleotides  ● Involved in neurotransmission and synaptic regeneration  ● Antioxidant activity  ● Stabilizing agent for increasing absorption of metal ions | [36], [37] |
| **B15 (pangamic acid)**  **Pubchem:** [**45934203**](https://pubchem.ncbi.nlm.nih.gov/compound/45934203) | **Undetermined RDA** | **Undetermined RDIs** | ● No reliable scientific evidence, given the enormous confusion regarding the identity of this vitamin. | [38], [39] |
| **B17 (amygdalin or laetrile)**  **Pubchem: 656516** | **Undetermined RDA** | **Undetermined RDIs** | ● First evidence of neuroprotective effects in acute ischemic stroke. | [40], [41] |
| **C**  **Pubchem:** [**54670067**](https://pubchem.ncbi.nlm.nih.gov/compound/54670067) | **PRI:**  **♂ : 110 mg/day**  **♀ : 95 mg/day** | **RDIs:**  **♂♀ : 90 mg/day** | ● Antioxidant activity.  ● Involved in neurotransmission  ● Synthesis of neurotransmitters, including serotonin and norepinephrine (NE).  ● Protective role against Aβ plaques formation and degradation. | [11], [42], [43], [44], [45], [46] |

**References**

[1] “Dietary Reference Values for the EU.” Accessed: Jul. 28, 2025. [Online]. Available: https://multimedia.efsa.europa.eu/drvs/index.htm

[2] “Nutrient Recommendations and Databases.” Accessed: Jul. 28, 2025. [Online]. Available: https://ods.od.nih.gov/HealthInformation/nutrientrecommendations.aspx#dritool

[3] O. F. Sultana, R. A. Hia, and P. H. Reddy, “A Combinational Therapy for Preventing and Delaying the Onset of Alzheimer’s Disease: A Focus on Probiotic and Vitamin Co-Supplementation,” *Antioxidants*, vol. 13, no. 2, p. 202, Feb. 2024, doi: 10.3390/antiox13020202.

[4] J. Almaguer, A. Hindle, and J. J. Lawrence, “The Contribution of Hippocampal All-Trans Retinoic Acid (ATRA) Deficiency to Alzheimer’s Disease: A Narrative Overview of ATRA-Dependent Gene Expression in Post-Mortem Hippocampal Tissue,” Nov. 01, 2023, *Multidisciplinary Digital Publishing Institute (MDPI)*. doi: 10.3390/antiox12111921.

[5] N. L. Nguyen, T. X. Hoang, and J. Y. Kim, “All-Trans Retinoic Acid-Induced Cell Surface Heat Shock Protein 90 Mediates Tau Protein Internalization and Degradation in Human Microglia,” *Mol Neurobiol*, vol. 62, no. 1, pp. 742–755, Jan. 2025, doi: 10.1007/s12035-024-04295-1.

[6] E. de Hoog, M. K. Lukewich, and G. E. Spencer, “Retinoic acid inhibits neuronal voltage-gated calcium channels,” *Cell Calcium*, vol. 72, pp. 51–61, Jun. 2018, doi: 10.1016/j.ceca.2018.02.001.

[7] M. Grimm *et al.*, “Vitamin D and Its Analogues Decrease Amyloid-β (Aβ) Formation and Increase Aβ-Degradation,” *Int J Mol Sci*, vol. 18, no. 12, p. 2764, Dec. 2017, doi: 10.3390/ijms18122764.

[8] C. Annweiler, “Vitamin D in dementia prevention,” *Ann N Y Acad Sci*, vol. 1367, no. 1, pp. 57–63, Mar. 2016, doi: 10.1111/nyas.13058.

[9] L. M. Huiberts and K. C. H. J. Smolders, “Effects of vitamin D on mood and sleep in the healthy population: Interpretations from the serotonergic pathway,” Feb. 01, 2021, *W.B. Saunders Ltd*. doi: 10.1016/j.smrv.2020.101379.

[10] R. P. Patrick and B. N. Ames, “Vitamin D hormone regulates serotonin synthesis. Part 1: Relevance for autism,” 2014, *FASEB*. doi: 10.1096/fj.13-246546.

[11] P. Mecocci *et al.*, “A Long Journey into Aging, Brain Aging, and Alzheimer’s Disease Following the Oxidative Stress Tracks,” 2018, *IOS Press*. doi: 10.3233/JAD-170732.

[12] S. O. Ekeuku, N. Mohd Murshid, S. N. Shukri, N. F. N. Mohd Sahardi, and S. Makpol, “Effect of Vitamin E on Transcriptomic Alterations in Alzheimer’s Disease,” Aug. 01, 2023, *Multidisciplinary Digital Publishing Institute (MDPI)*. doi: 10.3390/ijms241512372.

[13] M. Casati *et al.*, “Vitamin E and Alzheimer’s disease: the mediating role of cellular aging,” *Aging Clin Exp Res*, vol. 32, no. 3, pp. 459–464, Mar. 2020, doi: 10.1007/s40520-019-01209-3.

[14] N. A. Denisova and S. L. Booth, “Vitamin K and Sphingolipid Metabolism: Evidence to Date,” *Nutr Rev*, vol. 63, no. 4, pp. 111–121, Apr. 2005, doi: 10.1111/j.1753-4887.2005.tb00129.x.

[15] G. Ferland, “Vitamin K and the Nervous System: An Overview of its Actions,” *Advances in Nutrition*, vol. 3, no. 2, pp. 204–212, Mar. 2012, doi: 10.3945/an.111.001784.

[16] E. Emekli-Alturfan and A. A. Alturfan, “The emerging relationship between vitamin K and neurodegenerative diseases: a review of current evidence,” Jan. 01, 2023, *Springer Science and Business Media B.V.* doi: 10.1007/s11033-022-07925-w.

[17] S. Baltrusch, “The Role of Neurotropic B Vitamins in Nerve Regeneration,” 2021, *Hindawi Limited*. doi: 10.1155/2021/9968228.

[18] M. Mrowicka, J. Mrowicki, G. Dragan, and I. Majsterek, “The importance of thiamine (vitamin B1) in humans,” Oct. 01, 2023, *Portland Press Ltd*. doi: 10.1042/BSR20230374.

[19] A. L. Tardy, E. Pouteau, D. Marquez, C. Yilmaz, and A. Scholey, “Vitamins and minerals for energy, fatigue and cognition: A narrative review of the biochemical and clinical evidence,” Jan. 01, 2020, *MDPI AG*. doi: 10.3390/nu12010228.

[20] D. O. Kennedy, “B vitamins and the brain: Mechanisms, dose and efficacy—A review,” Jan. 27, 2016, *MDPI AG*. doi: 10.3390/nu8020068.

[21] M. Fila, C. Chojnacki, J. Chojnacki, and J. Blasiak, “Nutrients to improve mitochondrial function to reduce brain energy deficit and oxidative stress in migraine,” Dec. 01, 2021, *MDPI*. doi: 10.3390/nu13124433.

[22] V. Gasperi, M. Sibilano, I. Savini, and M. V. Catani, “Niacin in the Central Nervous System: An Update of Biological Aspects and Clinical Applications,” *Int J Mol Sci*, vol. 20, no. 4, p. 974, Feb. 2019, doi: 10.3390/ijms20040974.

[23] J. K. Blusztajn, B. E. Slack, and T. J. Mellott, “Neuroprotective actions of dietary choline,” Aug. 01, 2017, *MDPI AG*. doi: 10.3390/nu9080815.

[24] S. K. Tayebati and F. Amenta, “Choline-containing phospholipids: Relevance to brain functional pathways,” in *Clinical Chemistry and Laboratory Medicine*, Mar. 2013, pp. 513–521. doi: 10.1515/cclm-2012-0559.

[25] C. A. Calderón-Ospina and M. O. Nava-Mesa, “B Vitamins in the nervous system: Current knowledge of the biochemical modes of action and synergies of thiamine, pyridoxine, and cobalamin,” Jan. 01, 2020, *Blackwell Publishing Ltd*. doi: 10.1111/cns.13207.

[26] B. L. Zaric, M. Obradovic, V. Bajic, M. A. Haidara, M. Jovanovic, and E. R. Isenovic, “Homocysteine and Hyperhomocysteinaemia,” *Curr Med Chem*, vol. 26, no. 16, pp. 2948–2961, Mar. 2018, doi: 10.2174/0929867325666180313105949.

[27] T. Liwinski and U. E. Lang, “Folate and Its Significance in Depressive Disorders and Suicidality: A Comprehensive Narrative Review,” Sep. 01, 2023, *Multidisciplinary Digital Publishing Institute (MDPI)*. doi: 10.3390/nu15173859.

[28] P. J. Stover, J. Durga, and M. S. Field, “Folate nutrition and blood–brain barrier dysfunction,” Apr. 01, 2017, *Elsevier Ltd*. doi: 10.1016/j.copbio.2017.01.006.

[29] M. Yuasa *et al.*, “Effects of Biotin Deficiency on Biotinylated Proteins and Biotin-Related Genes in the Rat Brain,” 2016.

[30] C. E. Karachaliou and E. Livaniou, “Biotin Homeostasis and Human Disorders: Recent Findings and Perspectives,” Jun. 01, 2024, *Multidisciplinary Digital Publishing Institute (MDPI)*. doi: 10.3390/ijms25126578.

[31] A. J. López-Gambero, C. Sanjuan, P. J. Serrano-Castro, J. Suárez, and F. Rodríguez de Fonseca, “The Biomedical Uses of Inositols: A Nutraceutical Approach to Metabolic Dysfunction in Aging and Neurodegenerative Diseases,” *Biomedicines*, vol. 8, no. 9, p. 295, Aug. 2020, doi: 10.3390/biomedicines8090295.

[32] M. Krátký *et al.*, “4-aminobenzoic acid derivatives: Converting folate precursor to antimicrobial and cytotoxic agents,” *Biomolecules*, vol. 10, no. 1, Jan. 2020, doi: 10.3390/biom10010009.

[33] S. Venkatramanan, I. E. Armata, B. J. Strupp, and J. L. Finkelstein, “Vitamin B-12 and cognition in children,” 2016, *American Society for Nutrition*. doi: 10.3945/an.115.012021.

[34] A. R. Mathew *et al.*, “Vitamin B12 Deficiency and the Nervous System: Beyond Metabolic Decompensation—Comparing Biological Models and Gaining New Insights into Molecular and Cellular Mechanisms,” Jan. 01, 2024, *Multidisciplinary Digital Publishing Institute (MDPI)*. doi: 10.3390/ijms25010590.

[35] A. R. Mathew *et al.*, “Vitamin B12 Deficiency and the Nervous System: Beyond Metabolic Decompensation—Comparing Biological Models and Gaining New Insights into Molecular and Cellular Mechanisms,” *Int J Mol Sci*, vol. 25, no. 1, p. 590, Jan. 2024, doi: 10.3390/ijms25010590.

[36] C. Schiopu *et al.*, “Magnesium Orotate and the Microbiome–Gut–Brain Axis Modulation: New Approaches in Psychological Comorbidities of Gastrointestinal Functional Disorders,” Apr. 01, 2022, *MDPI*. doi: 10.3390/nu14081567.

[37] M. Löffler, E. A. Carrey, and E. Zameitat, “Orotic Acid, More Than Just an Intermediate of Pyrimidine de novo Synthesis,” 2015, *Institute of Genetics and Developmental Biology*. doi: 10.1016/j.jgg.2015.04.001.

[38] W. N. French and L. Levi, “Pangamic acid (vitamin B 15, pangametin, sopangamine): Its composition and determination in pharmaceutical dosage forms.,” *Can Med Assoc J*, vol. 94, no. 22, pp. 1185–7, May 1966.

[39] D. Schneider, V. Helwig, K. Staniek, H. Nohl, and E. F. Elstner, “Studies on the chemical identity and biological functions of pangamic acid.,” *Arzneimittelforschung*, vol. 49, no. 4, pp. 335–43, Apr. 1999.

[40] K. Kimura, Y. H. Liu, and C. L. Hsieh, “Amygdalin’s neuroprotective effects on acute ischemic stroke in rats,” *J Ethnopharmacol*, vol. 345, Apr. 2025, doi: 10.1016/j.jep.2025.119621.

[41] Y. She *et al.*, “Neuroprotective effect of glycosides in Buyang Huanwu Decoction on pyroptosis following cerebral ischemia-reperfusion injury in rats,” *J Ethnopharmacol*, vol. 242, Oct. 2019, doi: 10.1016/j.jep.2019.112051.

[42] F. Monacelli, E. Acquarone, C. Giannotti, R. Borghi, and A. Nencioni, “Vitamin C, aging and Alzheimer’s disease,” Jul. 01, 2017, *MDPI AG*. doi: 10.3390/nu9070670.

[43] L. Zylinska, M. Lisek, F. Guo, and T. Boczek, “Vitamin C Modes of Action in Calcium-Involved Signaling in the Brain,” Feb. 01, 2023, *MDPI*. doi: 10.3390/antiox12020231.

[44] M. E. Meredith and J. M. May, “Regulation of embryonic neurotransmitter and tyrosine hydroxylase protein levels by ascorbic acid,” *Brain Res*, vol. 1539, pp. 7–14, Nov. 2013, doi: 10.1016/j.brainres.2013.09.040.

[45] I. Sampaio, F. D. Quatroni, P. M. Pincela Lins, A. S. Nascimento, and V. Zucolotto, “Modulation of beta-amyloid aggregation using ascorbic acid,” *Biochimie*, vol. 200, pp. 36–43, Sep. 2022, doi: 10.1016/j.biochi.2022.05.006.

[46] “Scientific Opinion on Dietary Reference Values for vitamin C,” *EFSA Journal*, vol. 11, no. 11, Nov. 2013, doi: 10.2903/j.efsa.2013.3418.
